# Ultra-compact plasma photocathode in a hybrid wakefield accelerator

**DOI:** 10.1038/s42005-026-02814-1

**Published:** 2026-08-17

**Authors:** Patrick Ufer, Alastair Nutter, Susanne Schoebel, Thomas Heinemann, Brant Bowers, Finn-Ole Carstens, Yen-Yu Chang, Sébastien Corde, Jurjen Pieter Couperus Cabadağ, Alexander Debus, Andreas Döpp, F. Moritz Foerster, Max F. Gilljohann, Ahmad Fahim Habib, Stefan Karsch, Alexander Knetsch, Olena Kononenko, Maxwell LaBerge, Alberto Martinez de la Ossa, Richard Pausch, Ross Rudzinsky, Andrew Sutherland, Thomas Wilson, Arie Irman, Ulrich Schramm, Bernhard Hidding

**Affiliations:** 1 Helmholtz-Zentrum Dresden-Rossendorf, Dresden, Germany; 2 Technische Universität Dresden, Dresden, Germany; 3 University of Strathclyde, Glasgow, UK; 4 The Cockcroft Institute, Warrington, UK; 5 Heinrich Heine University Düsseldorf, Düsseldorf, Germany; 6 Department of Physics, University of Texas at Austin, Austin, TX USA; 7 LOA, ENSTA Paris, CNRS, Ecole Polytechnique, Institut Polytechnique de Paris, Palaiseau, France; 8 Ludwig-Maximilians-Universität München, Garching, Germany; 9 Max Planck Institut für Quantenoptik, Garching, Germany; 10 SLAC National Accelerator Laboratory, Menlo Park, CA USA; 11 Deutsches Elektronen-Synchrotron DESY, Hamburg, Germany

**Keywords:** Plasma-based accelerators, Plasma physics

## Abstract

Plasma-based particle accelerators, realized by driving plasma waves with either an intense laser pulse or particle beam, offer accelerating fields orders of magnitude higher than conventional accelerators. This enables sources of highly relativistic electron beams with significantly reduced spatial and economic footprints. However, optimizing beam quality and brightness remains the key challenge for demanding applications such as free-electron lasers. The plasma photocathode is a method for generating high-quality beams directly within plasma waves - but hitherto was exclusively realizable using the high-current drive beams from the kilometer-scale conventional accelerator at SLAC. Here we show implementation of a plasma photocathode in a millimeter-scale, purely plasma-based hybrid wakefield accelerator, powered by a 150 TW laser system. Its operation at high plasma densities enables exploitation of micrometer-scale source sizes and rapid acceleration through high-field gradients, which increases the potential output beam quality substantially. This high-density plasma photocathode can be implemented at state-of-the-art laser-plasma accelerators worldwide, and opens the door for high-quality beam production.

## Abstract (web summary)

The two-stage hybrid plasma accelerator combines laser- and particle-driven acceleration to realize a compact source of relativistic electron bunches. Here, the authors demonstrate the implementation of plasma photocathode injection in such a hybrid platform, establishing a route toward the generation of low-emittance electron beams.

## Introduction

Radiofrequency cavity-based linear accelerators (linacs) are technologically limited to accelerating fields up to  ≈ 100 MVm^−1^^1^. This leads to large accelerator sizes, and constrains the quality and brightness of electron beams extracted from the cathode, as rapid acceleration of emitted electrons is crucial when optimizing for beam quality^2^. In linacs, photocathodes, which employ laser pulses to extract electrons from a mm-scale spot on the cathode, are the gold standard for beam quality.

Plasma-based accelerators can provide orders of magnitude larger electric fields than conventional accelerators^3–9^ by transient, collective separation of electrons from ions. In particle beam-driven plasma wakefield accelerators (PWFA), the Lorentz-contracted transverse fields of intense electron beams drive plasma electrons away from the much heavier, practically immobile ions^10^.

In the wake of the driver beam, the ions’ electric fields re-attract plasma electrons, forming an electron plasma wave whose cavities can be exploited for high-gradient acceleration^11^. The plasma density *n*_e_ determines a number of parameters, such as the peak obtainable wakefield amplitude $E\propto \sqrt{{n}_{{{{\rm{e}}}}}}$ and the plasma wavelength ${\lambda }_{{{{\rm{p}}}}}\propto {n}_{{{{\rm{e}}}}}^{-1/2}$. However, driving high-amplitude waves in dense plasmas requires short, high-current drive beams, which are difficult to generate using conventional accelerators. Another challenge is generating high-quality beams from such plasma waves^12^, with various methods being investigated^13–16^. The plasma photocathode approach employs a synchronized laser pulse to release additional electrons directly within the plasma wave, stripping them from higher ionization levels than those of the background plasma species^17^. Subsequently, these electrons are rapidly accelerated and focused by the plasma wakefields, forming a so-called witness beam. This mechanism could produce beams orders of magnitude brighter than the corresponding input driver beam^17^, surpassing those generated by state-of-the-art photocathode linacs^18^. It therefore has significant implications for applications such as plasma-based X-ray free-electron lasers^19–23^. However, generating high-quality beams requires operating in the so-called blowout regime, where the driver beam density *n*_b_ exceeds the plasma density *n*_e_^11^. In this regime, all plasma electrons are expelled by the driver, resulting in a plasma wave possessing a particularly high electrostatic potential, enabling the capture of electrons liberated at rest in the center of the plasma wave. Additionally, the wakefields are linear and hence capable of conserving high beam quality without brightness loss during subsequent acceleration (see Supplementary Note 1). To the best of our knowledge, only the kilometer-long SLAC FACET linac^6,7,24^ produced drive beams capable of driving PWFA in the blowout regime at plasma densities of *n*_e_ ≈ 1.3 × 10^17^ cm^−3^, resulting in plasma wavelengths *λ*_p_ ~ 100 μm and accelerating gradients *E* > 10 GV/m, suitable for plasma photocathode injection^13^.

To address the scarcity of sufficiently strong particle driver beams, an alternative approach was developed^9,25^: exploiting electron beams produced by laser-driven plasma wakefield accelerators (LWFA) to drive a subsequent PWFA stage. The short ( < 20 fs), high-current ( > 10 kA) scale bunches produced by LWFA^26^ enable PWFA operation in the blowout regime, with field amplitudes exceeding *E* ~ 100 GV/m, and plasma wavelengths around *λ*_p_ ~ 20 μm. This hybrid LWFA → PWFA method enables PWFA research on meter-scale footprints for laboratories worldwide. It has previously been successfully implemented in cutting-edge laser-plasma experiments, delivering significant advancements in PWFA research ^15,16,27,28^.

Here, we demonstrate the experimental realization of an ultracompact plasma photocathode integrated into, and synchronized with, a compact hybrid plasma wakefield accelerator. This regime of plasma photocathode benefits from transverse source sizes of the order of a few μm^2^, and rapid acceleration in the high-field blowout regime.

## Results

### Experimental setup

**Fig. 1:**
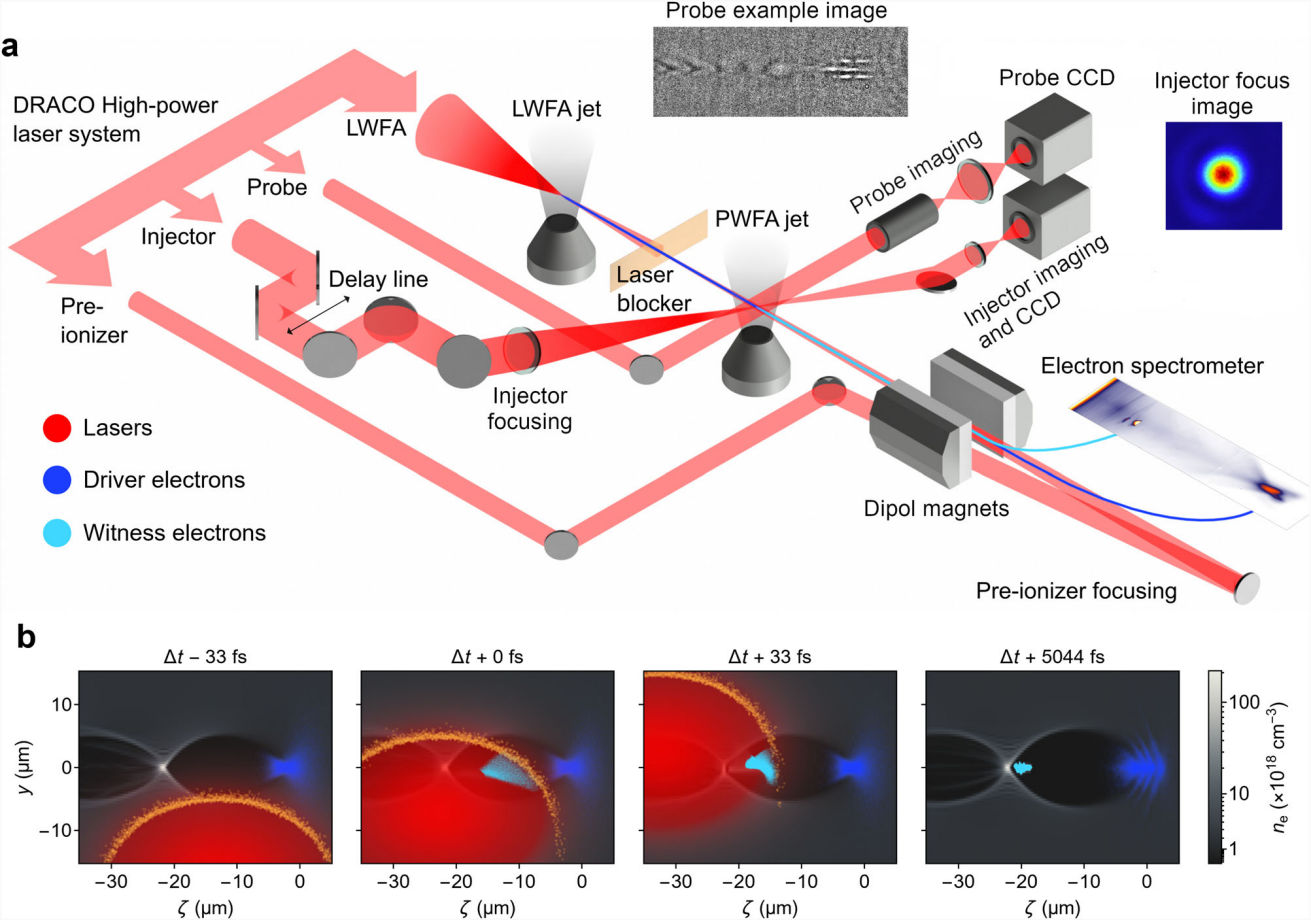
Experimental setup. **a** Overview of the experimental setup. All lasers (red) originate from the DRACO laser source. The LWFA laser produces an electron beam in the LWFA jet and is removed by the laser blocker foil (orange). The created electron beam (dark blue) is then propagated to the PWFA stage, which is already ionized by the pre-ionizing laser. The injector creates the witness beam (ligh blue) which is recorded together with the driver by the electron spectrometer. An image of the injector focus is shown in the inset on the right. A probe beam is used to view the interaction with an example image shown in the upper inset. **b** Snapshots from particle-in-cell simulations showing the injection process, with *ζ* as the co-moving frame propagation axis coordinate. The electron beam (dark blue) drives a plasma wave in the blowout regime, and the ionization front (orange) of the injector laser pulse (red) ionizes He^2+^ while sweeping across the plasma cavity. He^2+^ electrons liberated in the center of the plasma wave (light blue) are subsequently rapidly compressed, trapped and accelerated in the plasma wave, forming the witness beam.

The experiment is conducted using the DRACO laser at the Helmholtz-Zentrum Dresden-Rossendorf. This system provides four inherently synchronized laser beams, implemented in the experimental setup as shown in Fig. 1a). The main 150 TW laser pulse is focused onto the LWFA stage, which consists of a 3 mm supersonic gas nozzle providing high gas densities. This stage provides a relativistic, high peak-current electron beam of a few-femtosecond duration (see Methods). Following the LWFA stage is a thin laser blocker foil that reflects the spent LWFA laser pulse, preventing it from ionizing the PWFA stage.

The PWFA stage, also a 3 mm gas nozzle, provides a 50:50 hydrogen-helium gas jet, at a gas density *n* = 1.15( ± 0.05) × 10^18^ cm^−3^. A counter-propagating preionization laser pulse forms a wide plasma channel through partial ionization of this gas mixture (see Methods) prior to the arrival of the electron beam. Electrons are released from both hydrogen and the first ionization level of helium, with the remaining fraction reserved for plasma photocathode injection. The electron beam generated in the LWFA stage propagates through the blocker foil and drives a plasma wake in this preionized plasma channel.

A third laser pulse, utilized for injection, is focused to a full-width at half-maximum (FWHM) spot size *w*_0_ = 16.4 μm at 90° to the propagation axis (see Methods). It is centered on the injection point (IP), which is located 700 μm into the 3 mm long PWFA stage. It ionizes electrons from the second ionization level of helium, designated as the injection target species, inside the plasma wave in order to achieve witness beam production from the plasma photocathode process (see Fig. 1b). As shown, the laser ionization front is wider than the plasma wavelength, resulting in the entire blowout being filled with released electrons once overlap is achieved between the injector laser and plasma blowout. An example of the injector laser pulse’s vacuum transverse intensity profile is shown as inset in Fig. 1a).

Finally, a collimated few-cycle probe laser pulse (see Methods), also angled at 90 degrees to the electron beam propagation, is used to provide detailed shadowgraphy images of the region around the IP for diagnostic purposes. This method greatly benefits from the high plasma densities employed within the PWFA stage, as the density strongly correlates with image contrast^27^. Another key diagnostic is the electron spectrometer, which allows characterization and measurement of the accelerated beams (see Methods). This setup builds upon the hybrid LWFA → PWFA platform previously developed^9,15^, enhanced for the plasma photocathode injection scheme.

### Realizing the plasma photocathode

In order to realize the plasma photocathode, intensities of *I*_inj_ ≈ 1.5 × 10^17^ W/cm^2^ are utilized, ensuring full ionization of the remaining helium. In addition, the plasma photocathode relies on precise spatiotemporal alignment of the injector laser and plasma wake, facilitated through the use of the shadowgraphy images.

**Fig. 2:**
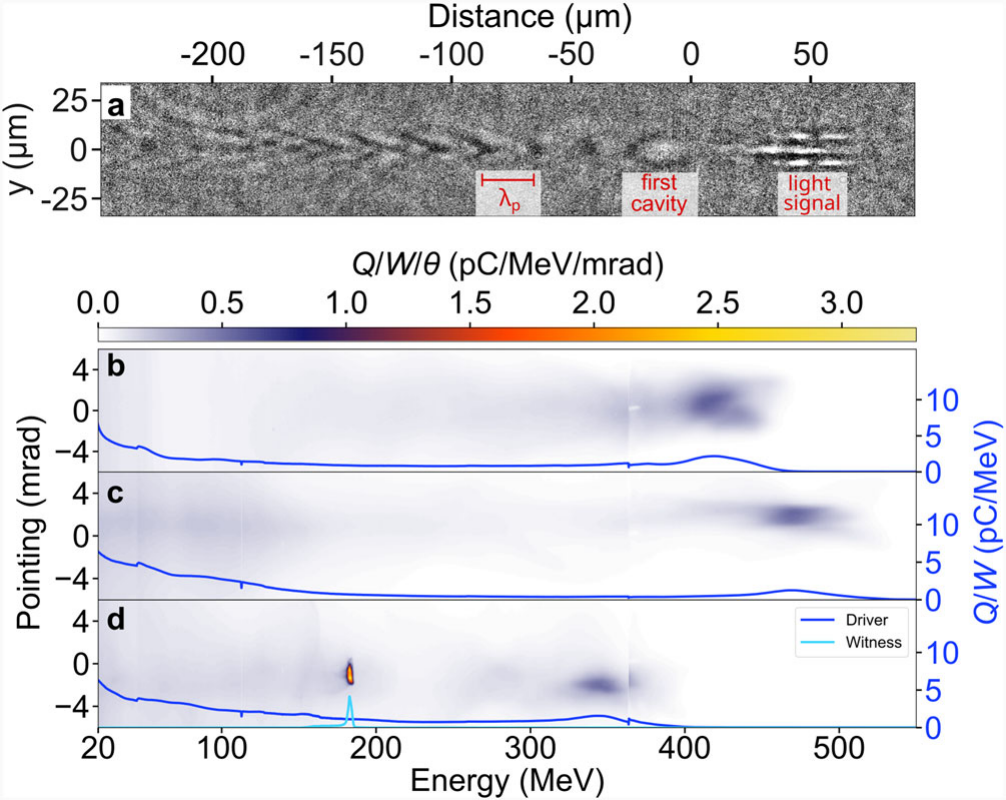
Shadowgraphy and electron spectrometer data. **a** Shadowgraphy of the injection point showing the plasma wake within the PWFA stage. **b** LWFA generated electron beam spectrum, with blocker tape. **c** LWFA generated electron beam spectrum including the PWFA stage and blocker tape, but without the injector laser. **d** LWFA generated electron beam spectrum including the PWFA stage, blocker tape and the injector laser, resulting in a clearly defined witness beam. The lineouts correspond to the spectral charge density of the beams as indicated.

An annotated example of the shadowgraphy is shown in Fig. 2a), and reveals that a pronounced plasma wave of *λ*_p_ ≈ 22 μm is driven by the electron driver beam. In addition, a light signal appears on the probe imaging system, typically manifesting with a three-stripe structure. The occurrence of this correlates with the spatiotemporal overlap between the injector laser and beam-driven wake, appearing at the position they intersect (see Supplementary Note 2). The combination of this unexpected tool with the few-cycle probe shadowgraphy enabled reliable overlap.

Figure 2b–d) provide energy spectra generated by the LWFA, the LWFA → PWFA without an injector laser, and the LWFA → PWFA with a spatiotemporally aligned injector laser, respectively, the latter presenting a clearly identifiable witness beam. The absence of a significant additional accelerated charge in Fig. 2c), aside from the electron driver beam, indicates that the PWFA operates in a practically dark-current-free regime, with no witness beam being injected.

The witness beam indicated in Fig. 2 d) has a charge of *Q* ≈ 10.7 pC within the full-width at quarter-maximum (FWQM), a root-mean-square (RMS) divergence of 0.59 mrad, and an energy spread of 1.24 MeV (RMS) with a mean energy of 183.1 MeV, resulting in a relative energy spread of only 0.68% (see Methods). In comparison to the drive beam, this high-quality witness beam exhibits drastically smaller energy spread and divergence, and a significant increase in spectral charge density.

### Investigating the plasma photocathode

**Fig. 3:**
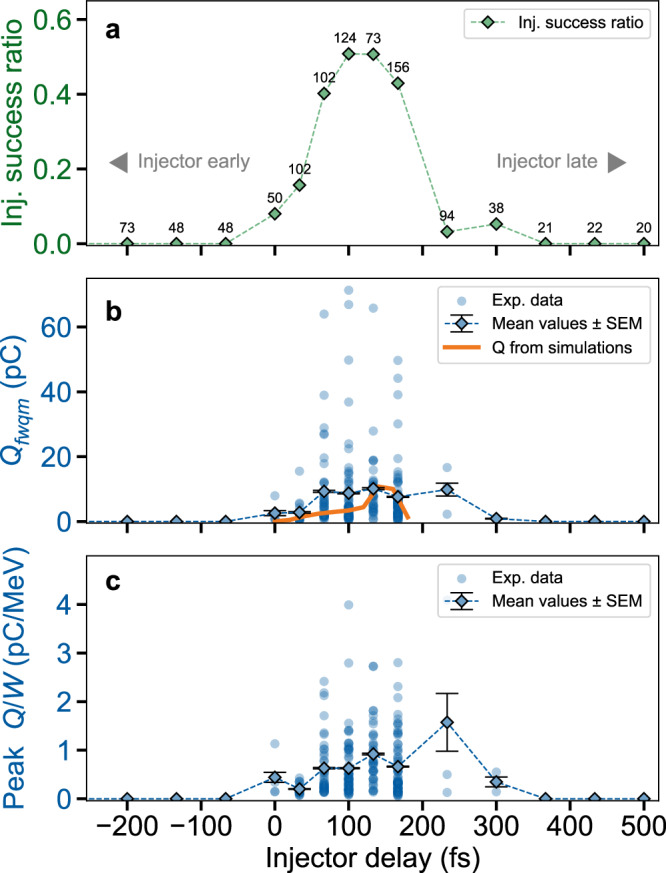
Injected charge timing window. **a** Experimentally observed injection success ratio with the number of total shots at each delay displayed above the data points. **b** Experimentally injected witness beam charge and charge observed in simulations. **c** Spectral charge density of injected witness beams. Individual shots are plotted with reduced opacity to indicate clustered charge values. The mean values ± SEM of the witness parameter are shown for each timing with the number of witnesses used for the analysis amounting to *N* = 4, 16, 41, 63, 37, 67, 3 and 2 for the delays of 0 fs to 300 fs, respectively. The delay of 0 fs is defined as the earliest timing where witness beams are observed. See also Supplementary Fig. 11.

To investigate the injection process, we determine the specific delay range between the injector laser and electron drive beam that enables successful witness beam generation, a key characteristic of plasma photocathode injection. The duration of this delay window is defined by the geometric overlap of the injector laser ionization front and the plasma wake. Simulation results (see below and Supplementary Note 6) indicate an expected delay window of approximately *τ*_delay_ ≈ 160 ± 10 fs across the first plasma cavity. Therefore, a scan of the injector laser time of arrival with respect to the electron drive beam was performed. Figure 3a) shows the injection success ratio; the number of shots with clearly observable witness beams divided by the total number of shots per delay. It indicates that the vast majority of shots with successful injection are confined to a timing range that is in good agreement with simulations and the expected timing window (see Supplementary Fig. 9).

If the helium is removed from the PWFA gas mixture, hence eliminating the injection target species, no witness beams are detected. Likewise, if the injector laser path is blocked, thereby preventing ionization of the injection target species, no witness beams are observed (see Supplementary Note 5 and Supplementary Fig. 1). The characteristic dependency on spatiotemporal alignment and the requirement for selective laser-triggered ionization give clear evidence that we succeeded in realizing plasma photocathode injection in this ultracompact setup.

Additional analysis of the witness beams is given in Fig. 3b), which displays the measured charge during the delay scan. The scan demonstrates that both the average and maximum attainable witness-beam charge depend on the injector-laser delay. This is the result of the changing degree of overlap between laser and plasma cavity, which defines how much charge is released within the cavity (see Supplementary Fig. 2). This correlation is a further characteristic signature of 90° plasma photocathode injection. The same witness beams are presented in Fig. 3c), which shows their spectral charge density obtained from the spectrometer, a metric used as a relative measure of beam quality. As is evident from Fig. 3, the experimentally observed witness beam charge fluctuates significantly from shot to shot.

Aside from the spatiotemporal jitters, analysis of the plasma wavelength via shadowgraphy reveals variance in the background plasma density (see Methods). This can be attributed to the premature ionization of the injection target species, the degree of which is affected by not only the preionization laser, but also shot-to-shot jitter in the electron drive beam parameters, particularly its charge. This premature depletion determines how much charge the injector laser can release within the wake, thereby limiting the maximum witness beam charge.

**Fig. 4:**
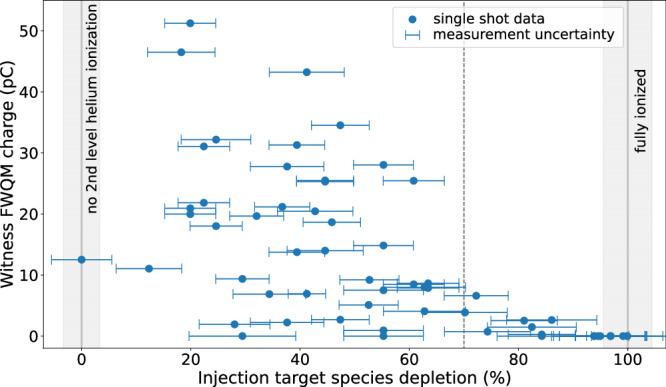
Witness beam charge dependency. Correlation between witness beam charge with injection target species ionization, determined through shadowgraphy analysis, from a separate dataset than the one presented in Fig. 3. The gray lines show the limits of 0 and 100% depletion of the injection target species, with their uncertainty indicated by the width of the shaded gray regions. The error bars are defined by the measurement uncertainty of the cavity length (two pixel accuracy) divided by the number of cavities used to determine the wavelength from which the depletion is derived and are determined for each measurement individually (*N* = 1). The additional dotted vertical line indicated at 70% depletion references the working point of the simulation studies shown in Fig. 3b) and in the Supplementary Note 6 and Supplementary Figs. 2, 4.

The jitter in the degree of depletion and its correlation with measured witness charge can be seen in Fig. 4. The data indicate that the degree of premature ionization of the injection target species ranges from negligible to total depletion. Additional analysis of the shadowgraphy captures an elongation of the first plasma cavity relative to the trailing wake, with the degree of elongation having been found to correlate with driver charge^28^. Variance in both the degree of ionization and elongation will strongly affect witness beam generation. These effects compound with jitter in the spatiotemporal alignment and lead to both the charge variance observed in Fig. 3b), and the spread of attained witness beam energies (see Supplementary Note 4, Supplementary Figs. 3, 11). Witness beams are observed at energies *W* > 250 MeV, which is consistent with accelerating field gradients of  ~ 100 GVm^−1^.

### Simulating high-density plasma photocathode

While measurements of these experimental observables provide global insight into the high-density plasma photocathode, we employ three-dimensional particle-in-cell (PIC) simulations to investigate hidden aspects of the injection process that are inaccessible to direct experimental observation. The simulations use input parameters representative of the experimental conditions (see Methods) and reproduce the experimentally observed timing window in good agreement (see Fig. 3b). To capture the influence of experimental fluctuations, additional simulations were performed across a broad parameter space, chosen to mimic the variance introduced by the aforementioned jitter sources (see Supplementary Note 6). These simulated scenarios reflect the range of experimentally observed projected beam properties. Notably, consistent features systematically emerge across the scanned simulation parameter space. At the core of these results lie the intrinsic dynamics of the plasma photocathode injection process at high plasma densities. The combination of compact release volumes, ultrafast release times, and rapid acceleration to relativistic velocities causes the released electrons to arrange themselves in well-defined, spatial slices of the witness beam. Electrons released in the center of the plasma cavity automatically form the witness bunch head and obtain the highest quality both in terms of slice emittances and slice energy spread, whereas electrons from farther outside the wake center form the tail of the beam with slightly reduced, yet still excellent quality (see Supplementary Figs. 4, 5). Whilst a full exploration of the available parameter space or optimizing output parameters such as slice emittance, is beyond the scope of this work, representative simulations using experimentally viable input parameters already yield average normalized slice emittance values as low as *ϵ*_n,s_ ~ 40 nm-rad (see Supplementary Note 6). At the same time, the beams exhibit a characteristic energy chirp.

## Discussion

This experiment achieved the realization of a high-density plasma photocathode injector, based on a purely laser-driven wakefield accelerator. Realizing a plasma photocathode with an LWFA drive beam has several advantages. First, it reduces the laboratory footprint from kilometer-scale conventional setups to mere meters, greatly lowering economic and infrastructural demands and placing plasma photocathodes within reach of many laser facilities worldwide. Second, using fs-short, LWFA-produced driver beams supports PWFA operation at high plasma densities. This high-density regime brings additional advantages. On the one hand, higher plasma densities reduce the blowout cavity size and thus the source volume of the injected electron beams. On the other hand, they enable high electric fields exceeding *E* > 100 GVm^−1^, as demonstrated by our setup, which rapidly accelerate and trap injected electrons, limit space-charge force-induced emittance growth during beam formation, and can preserve emittance due to the linear fields of the blowout regime. At the same time, the combination of the micrometer-sized blowout and perpendicular injection geometry ensures a release time on the order of tens of femtoseconds, thus limiting phase mixing of injected charge. Together, these properties are a recipe for high-quality electron beam generation. Third, the all-optical system provides driver beam-synchronized auxiliary lasers for injection and shadowgraphy, enabling femtosecond-level timing control and detailed insight into the plasma wave structure. The few-cycle laser shadowgraphy exploits improved contrast facilitated by the high-density regime, enabling spatiotemporal alignment of the various beams and permitting detailed analysis of the plasma wave.

Robustly measurable generated witness electron beam parameters like projected energy spread and divergence are already comparable with other high-quality beam generation studies^29–31^, and are a significant improvement over driver electron beam parameters. However, beam parameters vary strongly from shot to shot, and the best witness beams are observed as single-shot measurements. Shadowgraphic measurements reveal that these shot-to-shot variations correlate with driver-charge fluctuation-induced variations in the pre-ionization level and in the elongation of the first cavity. These measurements indicate that the drive beam prematurely depletes the high-ionization target species and is therefore too strong—a challenge diametrically opposed to linac-based PWFA, where reaching driver strengths sufficient to drive high-density plasma waves is very difficult. Lowering the driver strength, combined with ongoing advancements in LWFA stability, such as optimizing laser spatio-temporal properties, ensuring reliable long-term operation aided by machine-learning-based feedback systems, and utilizing steady, turbulence-free plasma sources from gas cells—is anticipated to improve reproducibility in future experiments. These stability efforts are shared objectives with the successful approach of directly LWFA-driven plasma-based free-electron lasers^32,33^.

Simulations reveal the systematic nature of the plasma photocathode injection mechanism in this regime. The simulations suggest that the slice brightness of beams produced in this configuration may exceed the state of the art by several orders of magnitude. However, slice parameters like emittance and energy spread are significantly more difficult to measure than projected ones, and beams with very different slice parameters may exhibit the same projected beam parameters (See Supplementary Note 7). Yet, it is specifically these slice beam properties that play a critical role in enabling important applications like free-electron lasers (see Supplementary Fig. 7), where achieving sufficiently low slice emittance and energy spread becomes increasingly difficult when progressing towards shorter FEL wavelengths^23^.

At present, diagnostic tools are not capable of resolving slice parameters at the precision required to validate such simulations. Consequently, accurate characterization of the witness beam’s projected and slice emittance, slice energy spreads, and (slice) current remains an important goal for future experiments. Achieving this will depend on the development of innovative diagnostic methods and technologies, as the direct measurement of slice brightness at the predicted levels currently exceeds the capabilities of even the most advanced light-source facilities, such as X-ray free-electron lasers^34^.

Our plasma photocathode realization also overcomes several limitations encountered in the proof-of-concept experiment at SLAC^13^ (see Supplementary Note 8). Most notably, the larger beam sizes and lower plasma densities at SLAC fundamentally limited the production of beams with normalized emittance below the 1 μm-rad level. In contrast, operation in the high-density regime demonstrated here enables simulated normalized slice emittances in the 50 nm-rad range – even though experimental confirmation is not yet possible. The higher plasma densities also eliminate the need for long pre-ionized channels, simplifying the experimental setup considerably, while intrinsic laser synchronization provides precise control over the injection window—both critical advantages over linac-driven systems.

In summary, this realization of a high-density plasma photocathode in a compact, all-optical setup successfully combines the strengths of LWFA and PWFA technologies. It represents an important step toward the production of ultrabright electron beams and applications that crucially depend on minimized slice brightness, such as compact X-ray free-electron lasers.

## Methods

### Electron driver beam generation in LWFA stage

The DRACO 150 TW arm is used to generate the electron beam, reaching 2.25 J with 30 fs FWHM duration at an 800 nm central wavelength on target. The laser is focused by an f/20 off-axis parabolic mirror to a vacuum spot size of *w*_0_ ≈ 21 μm FWHM. The electron beam generation is similar to previous works^9,15,28^. A gas jet of 3 mm length delivered by a supersonic nozzle creates a gas profile with a plateau region reaching a total electron density of *n*_e,LWFA_ ≈ 4.4( ± 0.1) × 10^18^ cm^−3^ (see Supplementary Fig. 10). The gas mixture is composed of helium doped with 3% nitrogen and the laser vacuum focus position is set to 2 mm downstream from the nozzle center in order to produce the electron beam via self-truncated ionization injection^35^, as has been done previously^26,36^. The obtained electron bunches show a peak at (482 ± 40) MeV energy containing (99 ± 26) pC in the FWHM, with an energy spread of (70 ± 19) MeV averaged over 55 shots. The spectrum includes (425 ± 43) pC above 100 MeV. A bunch duration of (14.8 ± 1.6) fs has been measured in previous experiments^37,38^, with the same LWFA injection process and stage being utilized here. Following the LWFA stage, this beam propagates through 1 mm of vacuum before traversing the 25 μm thick Kapton tape laser-blocker foil (see Supplementary Fig. 8).

### Preionization of PWFA stage

The PWFA stage is located 1.8 mm downstream of the laser blocker foil and is also provided by a 3 mm supersonic gas nozzle. It is supplied with a 50/50 mixture of hydrogen and helium at a gas density of *n*_PWFA_ = 1.15( ± 0.05) × 10^18^ cm^−3^. In order to produce a wide homogeneous plasma channel prior to electron driver arrival, a counter-propagating laser pulse is used in similar fashion as in refs. ^9,15,28^ to assist plasma wake formation. It is derived from the DRACO laser and operated at an energy of 21.6 mJ with a duration of  ≈ 40 fs focused with an f/50 spherical mirror. The energy and focusing of the laser is chosen so that it would form a  > 100 μm wide plasma channel on target, and the intensity would be strong enough to ionize hydrogen and the first level of helium in vacuum propagation. The relatively large width of the plasma channel is chosen to ensure that the electron beam propagation is fully covered by plasma. This is a necessary precaution because the preionization laser is angled relative to the main axis to prevent damage to either beam line.

### Plasma photocathode laser

The plasma photocathode laser is also derived from the DRACO laser. It propagates perpendicular to the main axis, is focused on the electron beam's propagation axis, and is monitored by a camera positioned after the beam crosses the interaction point. Its pointing can be adjusted through the tip and tilt of the last mirror before the IP, with the timing being set by a delay line further upstream. The pulse, with an energy of 23.3 mJ and a duration of  ≈ 40 fs, is focused with an f/20 lens to a measured focal spot size of *w*_inj_ ≈ 16.4 μm FWHM. It will therefore reach an estimated peak intensity of around *I*_inj_ ≈ 1.5 × 10^17^ W/cm^2^, strong enough to ionize the second level of helium, thereby enabling injection via ionization of the injection target species. Such an intensity is chosen to ensure full ionization, regardless of any injection-laser degradation due to laser-plasma interactions prior to the IP. A pointing variance with a standard deviation of around 2 μm is observed, which is considerably smaller than the transverse extent of the wakefield, ensuring a stable alignment on every shot.

### Probe laser shadowgraphy

To generate the few-cycle probe beam, the central part of the LWFA driver beam is parasitically picked up by a half-inch diameter mirror. It is spectrally broadened in a 1 m long, neon-filled hollow-core fiber and recompressed to a pulse duration of about 10 fs. The beam is collimated to a diameter of 5 mm, containing 0.3 mJ of energy. The probe beam is sent through the target perpendicular to the electron propagation axis and is then imaged onto a camera using a telecentric microscope objective and an achromat. This results in a total resolution of 0.34 μm/px. The shadowgraphy images obtained this way are then background-corrected and normalized to a reference image taken without the PWFA stage, improving the visibility of the plasma wave without affecting the image itself. The plasma wavelength is measured by averaging over several trailing cavities, allowing the calculation of both the plasma density and degree of injection target species depletion, with the latter assuming a constant neutral gas density. It should be noted that the combined capabilities of few-cycle shadowgraphy and high-density operation are a unique feature of our setup to provide direct evidence of operation in the high-field PWFA blowout regime.

### Electron spectrometer and witness spectra analysis

Both the electron beam and witness spectra are measured via an electron spectrometer consisting of a 0.4 m-long B = 0.9T dipole magnet^39^, deflecting upwards onto calibrated LANEX screens. The emission from these screens is detected by multiple cameras, which are calibrated for specific energy ranges. These images are then precisely stitched together to create a continuous spectrum capable of detecting beams up to 1.2 GeV. The witness spectra is then obtained by careful background subtraction (see Supplementary Note 3 and Supplementary Fig. 6), and is used to extract all the given parameters of the witness beam. It varies strongly in peak height and in the extent of the low energy tail (See Supplementary Fig. 3). Therefore, the measure of charge in the range of the full-width-quarter-maximum (FWQM) is used, which includes all charge down to a value of the quarter of the maximum peak in the spectra. This allows a better representation of the charge amount in the witness than a typical FWHM value, without overemphasizing noise. The RMS energy spread and the divergence for the shot in Fig. 3. are determined by a Gaussian fitting of the energy spectra and the pointing distribution of the witness, respectively.

### Partice-in-cell simulations

The 3D particle-in-cell (PIC) codes VSim^40^ and PIConGPU^41^ are used to independently investigate the ultracompact plasma photocathode mechanism. For VSim simulations, which are shown in Figs. 1, 3, and Supplementary Figs. 2, 3, 5, a co-moving window with a Cartesian simulation grid of *N*_z_ × *N*_x_ × *N*_y_ = 800 × 136 × 136 ≈ 14.8 million cells with a cell size of 0.15 μm in the longitudinal and 0.35 μm in the transverse direction is employed. Current smoothing, VSim’s perfect dispersion module, absorbing boundaries and a split field approach is used similarly as in ref. ^22^. The background plasma medium consists of the 50/50 hydrogen and helium mixture as used in the experiment and is modeled with 1 macroparticle per cell (PPC). The levels of ionization are implemented from the results of ADK^42^ simulations of the preionization laser, resulting in both fully ionized hydrogen and the first level of helium. The second level of helium is additionally up to 70% ionized, with the remaining partially ionized portion implemented as a cold fluid. The witness electrons are sourced from ionization of this remnant and are modeled with 8 macroparticles per cell to better resolve injected charge characteristics. The electron driver beam is implemented as a tri-Gaussian with 32 macroparticles per cell, across a range of charge and length values. The driver beam used to reproduce the timing injection window in Fig. 3 has charge *Q* = 165 pC, beam length *σ*_z_ = 2.3 μm RMS, width *σ*_r_ = 2.5 μm RMS, and a divergence *θ*_xy_ = 1.6 mrad RMS. These parameters are derived from measurements of the experimental driver beam. The plasma photocathode injector is implemented as a tri-Gaussian envelope laser pulse. The laser electromagnetic fields do not interact with the background plasma and are solely used as input for the ionization routines, with laser ionized charge being released as cold electrons. Parameters are taken from experimental measurements, and are set at an energy of 23 mJ, a laser waist of 14 μm and an estimated beam duration of 40 fs.

In order to validate the findings of these VSim simulations without relying on the ionization laser model, large-scale PIConGPU simulations are performed. These reproduce the findings of the VSim simulations not only qualitatively, but with respect to slice emittance (see Supplementary Note 6 and 7) also quantitatively. To ab-inito model the plasma photocathode laser, its wavelength needs to be resolved, leading to a simulation box size of *N*_*z*_ × *N*_*x*_ × *N*_*y*_ = 1536 × 768 × 128 with a cell size of *Δ*_*z*_ × *Δ*_*x*_ × *Δ*_*y*_ = 146 nm × 50 nm × 364 nm, a time resolution of *Δ*_*t*_ = 1.22 fs, polarized in the horizontal *z*-direction. Ionization is modeled using a combined BSI^43^ and ADK^42^ method. The plasma is initialized similarly to the VSim simulations, but with 2 particles per cell. The driver bunch is modeled equally to the VSim setup. The electromagnetic fields are modeled using an 8th-order finite difference time domain field solver as described in ref. ^44^ and the Esirkepov current deposition scheme^45^. The particle motion is modeled using the Boris method^46^.

This allows for the cross-check that the kinetic (ponderomotive) kick by the injector laser does not significantly impact the emittance of the injected beam, which can be ruled out as a result of these additional PIConGPU simulations.

## Supplementary information

Supplemental Material

## Data Availability

The data that support the plots within this paper and other findings of this study are available from the corresponding authors upon request.

## Code availability

The computer code used for processing the data presented in this paper is available from the corresponding author on request.
