# Supplementary material for: Ultra-compact plasma photocathode in a hybrid wakefield accelerator
Source: Commun Phys. 2026 Aug 17;9(1):273. doi: 10.1038/s42005-026-02814-1 (PMC13481246; doi:10.1038/s42005-026-02814-1)
Supplement: Supplementary file 1 [file 42005_2026_2814_MOESM1_ESM.pdf]

Supplementary Information

Supplementary Note 1: The high-field blowout regime and beam quality preservation

The blowout regime of PWFA [1, 2] is as desirable as it is difficult to reach. It constitutes a plasma wave, where all plasma electrons are pushed off axis by the driver beam, and a subsequent electron free ion cavity is formed behind the driver beam. This ensures maximized electric fields within the blowout structure, which can be exploited for maximized acceleration gradients. Furthermore, both the longitudinal as well as transverse electric fields within this blowout structure are linear. A consequence of Liouville's theorem for accelerator physics is that a beam's phase-space, and hence its normalized emittance ϵ_n and brightness can only be preserved when the transverse focusing fields during the acceleration and transport are linear. This is important for any accelerator due to the importance of emittance and brightness for key applications such as free-electron lasers and other beam-based light sources, as well as for high field physics and luminosity considerations in colliders. Emittance growth during acceleration is already a concern when the initial witness beam emittance is of the order of $\epsilon_\mathrm{n} \sim 1\,\mu\mathrm{m\,rad}$ as realized in PWFA so far, driven by particle beams from conventional accelerators [3–6]. For witness beams of $\epsilon_\mathrm{n} \sim 10\,\mathrm{nm\,rad}$-scale normalized emittance, as predicted by theory and simulations to be within reach of plasma photocathodes, emittance preservation on the single nm rad level will become crucial [7]. In other words, while weaker plasma waves and significant emittance growth may be tolerable for other types of beams and applications, operation in the blowout regime becomes essential for plasma photocathodes when optimizing for beam quality.

In order to drive a plasma wave in the blowout regime, the electron drive beam density n_b must first be greater than the background plasma density n_e. Additionally, it must be focused tightly such that its transverse extent σ_r obeys $\sigma_\mathrm{r} k_\mathrm{p} \ll 1$, with the plasma wave number $k_\mathrm{p} = 2\pi/\lambda_\mathrm{p}$. Finally, the drive beam length σ_z, must be shorter than the plasma wavelength whereby $\sigma_\mathrm{z} < 2k_\mathrm{p}^{-1}$ for resonant plasma wave excitation. The size and density of the LWFA-produced driver beams utilised in our experiment are compatible with operation in the blowout regime at plasma densities of approximately $n_\mathrm{e} > 10^{18}\,\mathrm{cm}^{-3}$. This is significantly beyond the capabilities of beams typically used for PWFA at even the largest conventional accelerators, where typically plasma densities in the range of $n_\mathrm{e} \approx 10^{15} - 10^{17}$ are employed [4–6, 8–11].

Operation at higher plasma densities yields smaller blowout sizes as well as stronger accelerating gradients. These field gradients result in rapid acceleration to relativistic energies for any injected witness beam. Rapid acceleration is crucial, as the impact of space-charge forces can result in transverse momentum growth during the formative phase of the beam, with the associated emittance growth degrading beam quality [12]. This effect scales with γ^{-2}, where $\gamma = 1 + W_\mathrm{kin}/W_0$ is the relativistic Lorentz factor between the kinetic energy W_kin and electron rest energy W_0.

Finally, the high-field blowout regime is also essential because it results in the plasma wave having a large electrostatic potential. This facilitates electrons released by plasma photocathodes within the plasma wave to gain kinetic energy quickly enough

to catch up with the wave's velocity and be trapped at the end of the blowout. Weaker plasma waves do not possess this ability.

In summary, the high-field blowout regime is essential for plasma photocathodes because of three key aspects. It allows capturing and trapping of electrons released by plasma photocathodes, it facilitates rapid acceleration and suppression of space-charge based emittance growth, and it facilitates preservation of emittance due to the linearity of the wakefields. Since hybrid LWFA→PWFA allows operation in the desired blowout regime of PWFA, and because the plasma photocathode laser pulse can in such systems be intrinsically synchronized with the driver laser and electron beam, this combination is therefore ideally suited for hosting plasma photocathodes.

Supplementary Note 2: Spatiotemporal overlap light signal

A characteristic light signal is captured by the imaging system of the probe laser and appears only when the injector laser is both present, and spatially ($\approx$ few μm) and temporally ($\approx$ few fs) aligned with the wakefield of the drive beam. It also occurs without the probe laser and when no injection is present (e.g. pure hydrogen is used). It is strongest when the injector laser hits the first plasma wave cavity, becoming significantly weaker if it instead hits any subsequent wave cavities. This observation leads to a preliminary hypothesis that the source of the signal is injector laser light scattering off of the electron sheath of the plasma wake, but the details of this process still require investigation. Despite these unknowns, and whilst not proof of injection, the signal is an intrinsic experimental signature of the proper injection constellation and can be harnessed as a useful auxiliary diagnostic without additions to the experimental setup.

Supplementary Note 3: Witness background subtraction

The overlap of the drive beam and the witness beam spectra makes background subtraction necessary to ensure accurate parameter extraction. The background subtraction makes use of the regular appearance of the drive beam at the lower energy range, where the witness beams are detected. Its functionality is shown in Extended Data Fig. 6. After the energy of the peak of the witness beam is determined (black dashed line), the profile of the background is fitted at just slightly higher energies than the peak of the witness (blue dashed line). This fit is thereafter used to determine the background over the whole energy range of the witness. In order to include background variances along the energy axis, a constant offset is determined by looking at background profile at divergences outside of the divergence range of the witness (red dashed lines). The measured spectra (Extended Data Fig. 6 a) can in most of the cases be precisely split into its two parts of the driver spectra and the witness spectra, especially with the witnesses that are very defined with a sharp peak in the angular spectral charge density.

Supplementary Note 4: Experimental witness beam energy

As shown in Fig. 3, the parameters of the witnesses varied strongly even when using the same experimental timing setting. As observed by shadowgraphy and explained in the main text, this can be attributed to shot-to-shot jitter e.g. of the driver beam

from the LWFA stage, which leads to not only differing degrees of target species depletion (see Fig. 4), but also to varying accelerating wakefield amplitudes. Extended Data Fig. 3 shows the variance in the witness energy spectra for one experimental setting, following background subtraction. The values for the mean energy fluctuate in a roughly 100 MeV wide window around an average of 145.4 MeV, consistent with acceleration gradients of ≈ 100 GVm^{-1}, with some witness beams achieving energies in excess of 200 MeV. Although the witness beams typically exhibit a sharp peak followed by varying degrees of a lower energy tail, one can clearly see the variance in the appearance of the spectra, which is reflected by the previously shown instabilities in parameters such as charge and spectral charge density. Summarizing, several factors influence the witness beam parameters. Some arise from fluctuations in the driver charge distribution and size and its effect on the wakefield, e.g. field strength, wakefield elongation and target species depletion. Additionally, pointing jitter between the driver beam and the injector laser can lead to different injection behaviors from shot to shot due to varying degrees of overlap. Further work is now required to reduce the impact of these jitter sources, and to explore the sensitivity of the injection process, in order to optimize both witness beam stability and quality.

Supplementary Note 5: Investigating the injection requirements

As alluded to in the main text, a number of additional tests are conducted to ascertain any critical injection dependencies. The first of these involve introducing a ceramic plate into the beamline of the injector laser, thereby preventing it from interacting with the PWFA stage. This test is shown in Ext. Data. Fig. 1, and clearly shows that when the injector laser is blocked in such a way, no witness beam generation is observed. Second, following spatiotemporal alignment and subsequent witness beam production, the gas supply for the PWFA stage is altered to instead provide pure hydrogen, thereby removing the injection target species. With this change, the preionization laser fully ionizes the PWFA stage prior to the arrival of the drive beam and injector, and as a result no witness beams are detected. Both of these results provide conclusive evidence that the injection scheme depends upon the injector laser pulse ionizing an injection target species. This aspect, combined with the identified delay window in the main manuscript, solidify the conclusion that injection is exclusively facilitated by the plasma photocathode mechanism.

Supplementary Note 6: PIC simulation investigation of the new Plasma Photocathode regime

Simulations allow one to investigate details of the ultracompact plasma photocathode mechanism. First, the shadowgraphy has shown that a significant range of driver beam current densities are sampled during the experiment. As a result, not only does the plasma wave size vary, but the level of depletion of the He^{2+} ionization level also, as evident from Fig. 4. Varying current densities can be the result of different charge values contained in the quasi-monoenergetic main portion of the electron beam, its length, shape, emittance, etc. [13]. For Extended Data Figs. 2, 4 and 5, the same driver beam is used as the one used for Fig. 3 b, and the degree of background He^{2+}

preionization is also set to the same value of 70%. To explore the size and shape of the plasma photocathode source volume, the produced witness beam macroparticles are tracked back from the end of the simulation to their origin. Extended Data Fig. 2 shows the injection source volume of beams from the plasma photocathode revealed in this way, across the full timing window of injection. This analysis reveals that for all timings between plasma wave and plasma photocathode laser, the source is very small and has transverse widths of a few μm, only. For particularly late or early injection times, the overlap between the plasma photocathode ionization front and the wake is reduced. This reduces the volume from which released electrons will be captured by the wake. This is consistent with the experimental observation of reduced witness beam charge for late or early timings (see Fig. 3).

The injection source volume transverse size is a small subset of the already small plasma cavity, creating an ideal condition for achieving low emittance. One may draw a parallel to photocathode sources in linacs, where likewise a small laser spot size on the photocathode is desirable to release electrons compactly in both real and phase space [14]. However, in state-of-the-art linac photocathodes, the laser spot size is of the order of mm, and the release time of the order of many picoseconds.

Following this finding, simulations also allow investigation of the slice composition of the producible witness beams. Extended Data Fig. 4 a) shows energy W, current I, normalized emittance $\epsilon_{\rm n}$ in both transverse planes, and the absolute and relative energy spread ΔW, obtained from the witness beam at the end of the PWFA stage. Three observations are striking: First, the beam has a pronounced and approximately linear energy chirp, the head of the beam having lower energy than the tail. Second, the head of the beam in particular exhibits extraordinarily low values of slice emittance. The dashed line marks the $\epsilon_{\rm n} = 100\,{\rm nm\,rad}$ level. Such low values, in both planes, were previously thought unobtainable from perpendicular plasma photocathode geometry [15]. Third, the slice energy spread is extraordinarily low, again in particular within the head of the beam. The dashed line marks the $\Delta W = 0.5$ MeV level. The concomitant low slice emittance and energy spreads imply spectacularly high slice 6D-brightness

$$B_{\rm 6D} = \frac{I_{\rm p}}{\epsilon_{\rm n,x}\epsilon_{\rm n,y} \cdot 0.1\%\Delta W/W}. \quad (1)$$

In order to understand the injection mechanism further, we map several key characteristics of the injection source volumes to the produced witness beam. First, the colour-coding in Extended Data Fig. 4 c) reveals that electrons released closest to the wake centre (blue) are trapped at earlier co-moving positions ζ, forming the head of the witness beam and hence obtaining lowest energy (compare Extended Data Fig. 4 a), top panel). This is understandable, because the electrostatic potential of the plasma wave is greatest in the wake centre, therefore leading to the earliest trapping positions within the wake, where the longitudinal wakefield amplitude is lower than farther behind in the wake. Electrons released farther away (green-yellow-red) from the wake centre, in contrast, sample a weaker electrostatic potential and are trapped further behind in the wake. They therefore form the tail of the produced beam, where they are accelerated by a larger longitudinal electric field amplitude. This explains the pronounced chirp of the beam seen in Extended Data Fig. 4 a), top panel. Next,

in Extended Data Fig. 4 d) we map the average transverse momentum the released electrons obtain. Again, the colour-coding reveals that electrons released closest to the wake center obtain lowest transverse momentum, whereas electrons released further outside the center obtain somewhat larger transverse momentum. This can be understood from the fact that in the absence of the large ponderomotive potential due to the laser kick, the transverse wakefield, which is zero on axis and in the blowout regime increases linearly with transverse distance from axis, determines the transverse Coloumb force and hence momentum. Finally, in Extended Data Fig. 4 b) the release time is mapped, from which two relevant observations arise. First, the overall release duration of trapped electrons is very short, amounting to less than 100 fs. Second, the release sequence is orderly due to the sweeping ionization front, which means electrons from co-located release regions are liberated within a fraction of the overall release duration. Since co-located release regions also produce co-located slices, as evident from Extended Data Fig. 4 a) and c), this means that electrons in co-located slices are produced ultrafast. Both the short overall release time and the ultrafast slice production minimize phase-mixing.

The small injection source volume size, small transverse momentum and limited phase mixing explain why the overall emittance and energy spread of the produced witness beams is so low, and also why they are especially low at the head of the witness beam. First, the positions x and momenta p_x (same holds for the y-plane) contribute to the normalized transverse (slice) emittance as

$$\epsilon_{\mathrm{n,rms}} = \frac{1}{m_0 c}\sqrt{\langle x^2\rangle\langle p_x^2\rangle - \langle x p_x\rangle^2} \tag{2}$$

Generally, the small range of transverse positions, the small range of transverse momenta assumed by released electrons, and the short release duration are a recipe for low emittance. Since slices at the front of the formed witness beam stem from particularly small transverse position ranges in the injection source volume, thus obtaining particularly small transverse momenta, and are released within a particularly short span of time, they exhibit lowest emittance. Electrons originating from farther outside, which form the subsequent slices and the tail of the witness beam, have larger emittance due to analoguous considerations. The short release duration also explains the small slice energy spread. While in [16] and with a collinear co-propagating plasma photocathode laser, the spot size and associated Rayleigh length determines the residual energy spread, here the ultrafast release means that electrons within one slice are born at nearly the same time especially for the head slices, hence sample the same acceleration history, and therefore obtain very similar energies. The residual energy spreads in this example are lower than even in the showcase used in [16] for the collinear plasma photocathode geometry.

While Extended Data Fig. 4 visualizes and explains details of bunch formation for one timing, Extended Data Fig. 5 shows that the same witness bunch formation mechanism is valid for the other timings, which were shown in Extended Data Fig. 2: in all cases, the witness beams exhibit the linear energy chirp, and the systematically low emittances and energy spreads particularly in the head of the beams. One inherent advantage of hybrid LWFA→PWFA photocathode is that the plasma photocathode

laser pulse is inherently synchronized to the electron beam and hence the plasma wave. Therefore one may select one timing as an operation point with good reproducibility.

However, as pointed out in the discussion, the shot-to-shot variation of the drive beam and the degree of ionization by drive beam and preionization laser, lead to significantly different plasma waves, as evident from shadowgraphy. As commented on in the Methods section, we explored stronger and weaker driver beams by varying both charge and duration. The corresponding driver beam density, and the corresponding beam evolution during propagation of the plasma also may vary the degree of $He^{2}+$ ionization, as shown in Fig. 4. Our simulation scans, which varied the total driver charge from 125 pC to 400 pC and the driver length from $2.3\,\mu$m to $9.2\,\mu$m RMS, indicate that the systematic mechanism described above is present across a wide range of corresponding driver currents. However, for too high driver beam densities the blowout is too strong, which spoils the systematic bunch formation process, and results in significant dark current generation. On the other hand, very low driver currents lead to a weaker and evolving wave, resulting in a decreased chirp and less trapped charge. Minimum average slice emittances amount to $\epsilon_{\mathrm{x}} \approx 66.5\,\mathrm{nm\,rad}$ and $\epsilon_{\mathrm{y}} \approx 40.4\,\mathrm{nm\,rad}$. While further numerical exploration is required, these simulations indicate that the discovered mechanism is present for a wide range of driver beam parameters, which is useful to plan experimental steps to validate and explore the injection mechanism and beam quality hypothesis further, and to make progress towards measuring the slice parameters and their evolution during beam extraction and transport.

Supplementary Note 7: Difference and importance of slice vs. projected beam parameters

Extended Data Fig. 7a,b visualizes the important distinction between slice beam parameters and projected beam parameters. As an example, the beam schematically shown in Extended Data Fig. 7a may have the same projected energy spread as the one shown in Extended Data Fig. 7b, while the beams possess completely different slice energy spreads. The same applies for other slice and projected characteristics, namely the transverse emittance in both transverse planes, and the current. Projected values are much easier to measure than slice properties. For example, the projected energy spread of a beam is a robust and straightforward observable by resolving the energy-dependent trajectories of electrons in a static magnetic field-based electron spectrometer. In contrast, to fully resolve slice energy spreads, one would desire an ultrafast diagnostics such as a transverse deflecting cavity – much more challenging devices [17] to design and operate by any standard, requiring radiofrequency cavity infrastructure. Additionally, resolving slice values becomes increasingly difficult the higher the energy of the beam is, and the shorter the beam is. To our knowledge, thus far no electron beam produced in a plasma accelerator was measured with a transverse deflecting cavity, and it is doubtful if the slice values of high-energy, femtosecond duration electron beams could be resolved even with today's most advanced transverse deflecting cavity technology. Measuring emittance is even more challenging – already for measuring projected values, let alone for slice-resolved measurements. This measurement difficulty of slice values of energy spread and emittance is in stark contrast with their importance. For example, in free-electron lasers, the electrons micro-bunch

and cooperate within slices of the characteristic cooperation length – if within this slice the energy spread and emittance of electrons is sufficiently small. Specifically, the electron the slice emittance must suffice $\epsilon_{\mathrm{n,slice}} < \lambda_{\mathrm{r}}\langle\gamma\rangle/4\pi$, where γ is the Lorentz factor associated with the energy of the electrons, and λ_{r} is the resonant FEL wavelength. Similarly, the slice energy spread must satisfy $\sigma_{\gamma,\mathrm{slice}} < \rho$, where ρ is the FEL Pierce parameter, $\sigma_{\gamma,\mathrm{slice}}/\gamma$ denotes the relative energy spread, which decreases as electron beam energy increases, and ϵ_{n} is the normalized emittance, which stays constant as energy increases. One could also write the emittance criterion in terms of the geometric slice emittance, which like the relative energy spread decreases with increasing electron energy and is related to the energy-normalized emittance through a factor of γ, as $\epsilon < \lambda_{\mathrm{r}}/4\pi$. Extended Data Fig. 7c visualizes this, and the well-known fundamental energy spread and emittance criteria. One therefore has to differentiate carefully between slice and projected, and normalized and relative (or geometric) values.

Supplementary Note 8: Comparison to the SLAC FACET proof-of-concept experiment

The ultracompact plasma photocathode presented here differs fundamentally from the earlier proof-of-concept demonstration at SLAC [18] in terms of the attainable beam quality. While in both experiments the plasma photocathode laser released electrons in a perpendicular geometry relative to the plasma-wave propagation axis, and the laser pulses had comparable focal spot sizes ($\sim 20\,\mu$m at SLAC vs. $\sim 16.4\,\mu$m at HZDR) and durations (65 fs FWHM at SLAC vs. 40 fs at HZDR), the dimensions of the driver beam and the plasma wave differ drastically between the two setups. These differences translate into completely distinct temporal and spatial scales for electron release and trapping. As a result, the effective release volume from which electrons are captured, as well as the release duration, are approximately an order of magnitude smaller in the ultracompact plasma photocathode realized at HZDR than in the SLAC experiment. Furthermore, at SLAC the electron driver beam extended into the ionization region, imparting a transverse momentum kick to the released electrons. This interaction fundamentally limited the achievable normalized emittance to values no better than approximately $\sim 1\,\mu$m-rad.

Reference [18] noted that, in principle, substantially lower emittances could be achieved even in a 90° geometry, if a much larger blowout region were combined with a much tighter plasma photocathode laser focus of the order of 3 µm rms, at least in one transverse plane. In the plasma photocathode now realized, in contrast, we found a diametral solution to the issue, and show that by 'going smaller' with the plasma wave size, emittances of few tens of nm-rad scale are possible, in both planes, and with much higher charge. In essence this is due to size: the ultracompact source size, and an ultrashort release time, which limits phase mixing, represent an experimental scenario that for the first time can allow normalized emittances of the order of few tens of nm-rad to be realized. In this scenario the laser spot size is larger than the plasma wave, a fundamentally different scenario to the one at SLAC.

Both types of plasma photocathodes have thus far been demonstrated only in pioneering, first-of-their-kind experiments. Naturally, the stability and reproducibility obtained during these initial proof-of-concept experiments therefore is limited, and

they have been carried out on completely different accelerator platforms. Nevertheless, both avenues share common optimization pathways. A primary path for improvement lies in enhancing the reproducibility of the PWFA, for example through stabilization of the driver beam, and more controlled preionization. Laser-electron beam timing is likewise important: here, the hybrid plasma photocathode offers distinct advantages over linac-driven systems, benefiting from superior intrinsic synchronization and the fact that, in the hybrid configuration, the laser pulse spot size is larger than the plasma wave, rather than smaller.

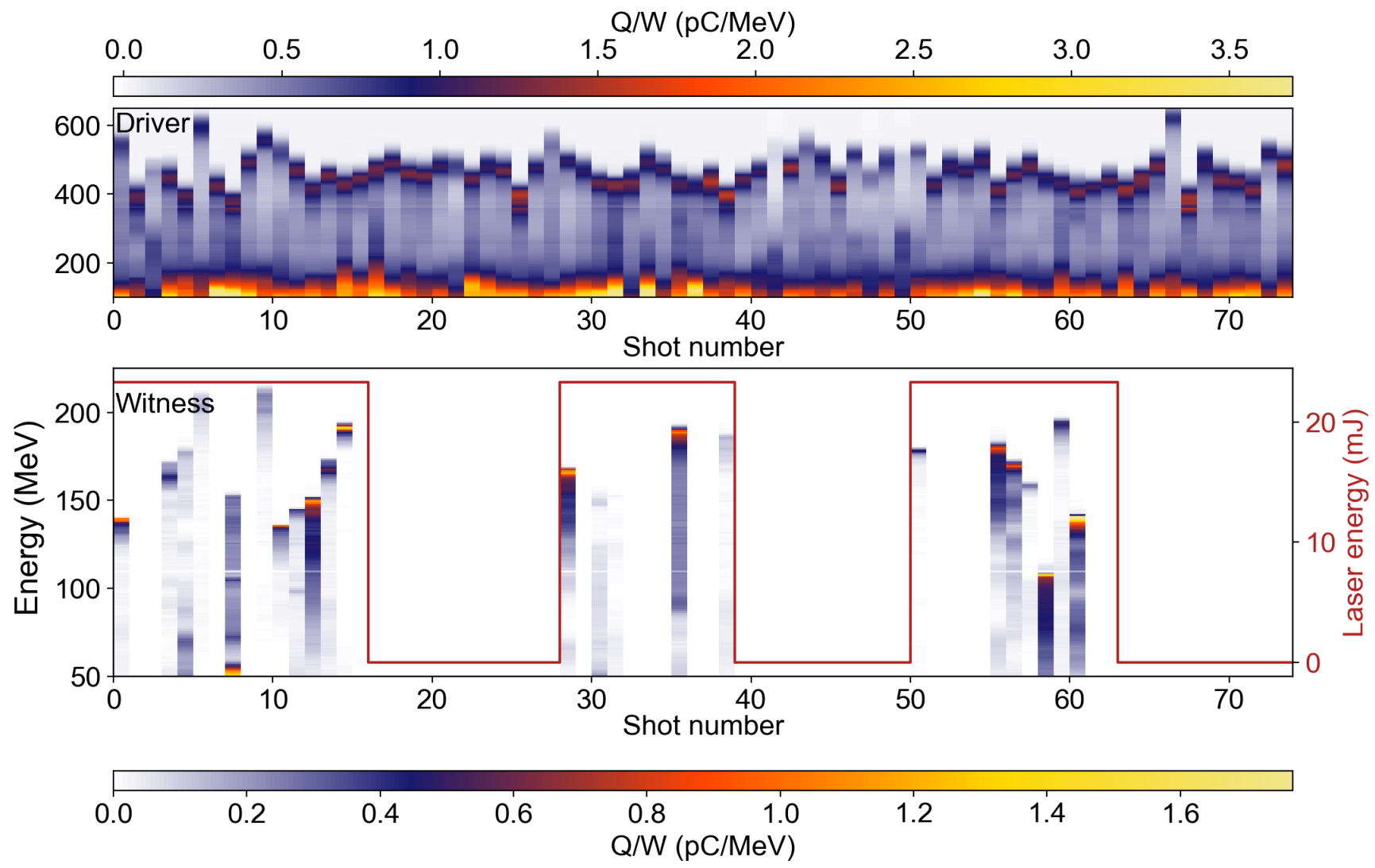

Supplementary Figure 1: Injector Laser On/Off-Scan. Measured spectra for witnesses (bottom) and corresponding driver (top) on consecutive shots with injector laser on and off indicated by its laser energy (red line), with a blocked laser indicated by a laser energy of 0 mJ.

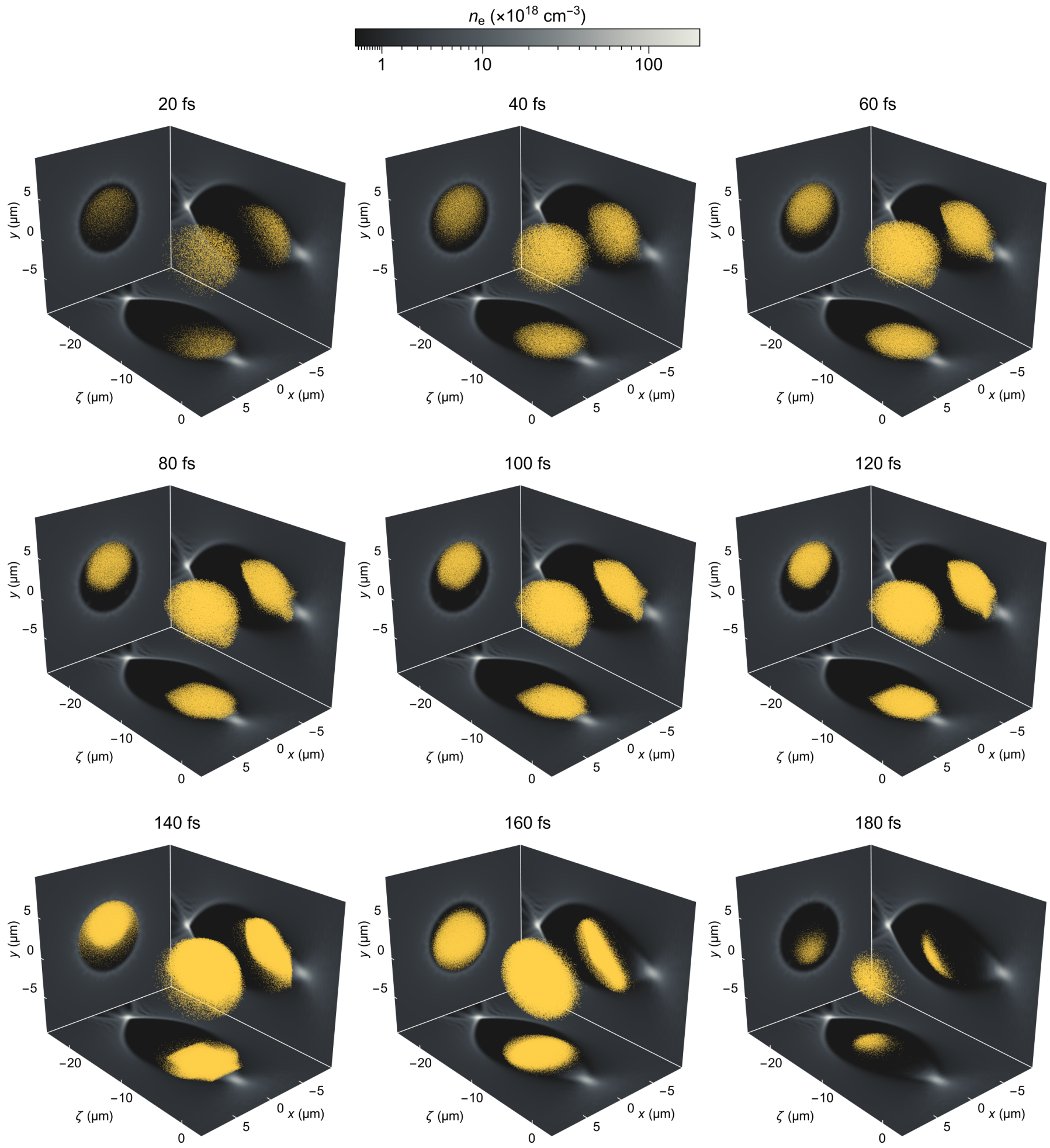

Supplementary Figure 2: Plasma photocathode source volumes obtained from 3D VSim PIC-simulations across the injection timing window. Electron macroparticles that form the witness beam are tracked back to their source to obtain the size and shape of the source from which electrons are successfully injected. The contour of the plasma wave at one point of time is shown in the projections, to indicate that the source volume is a subset from within the small plasma wave.

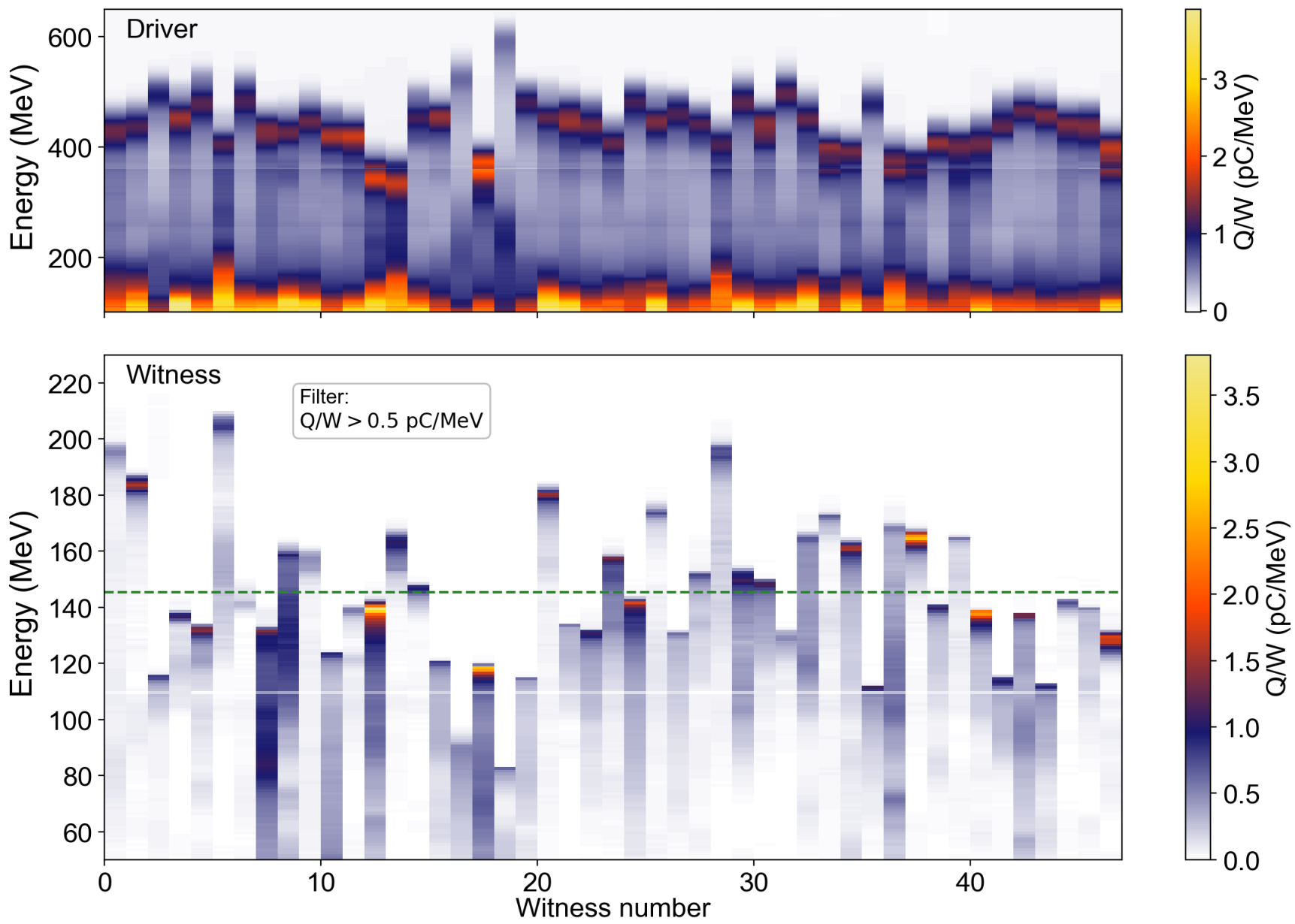

Supplementary Figure 3: Energy spectra of witnesses (bottom) separated from the driver (top) for witnesses of the timing 100 fs at the delay scan of Fig. 3 with spectral charge densities above 0.5 pC/MeV. The average of the witness mean energy is indicated with a green dashed line.

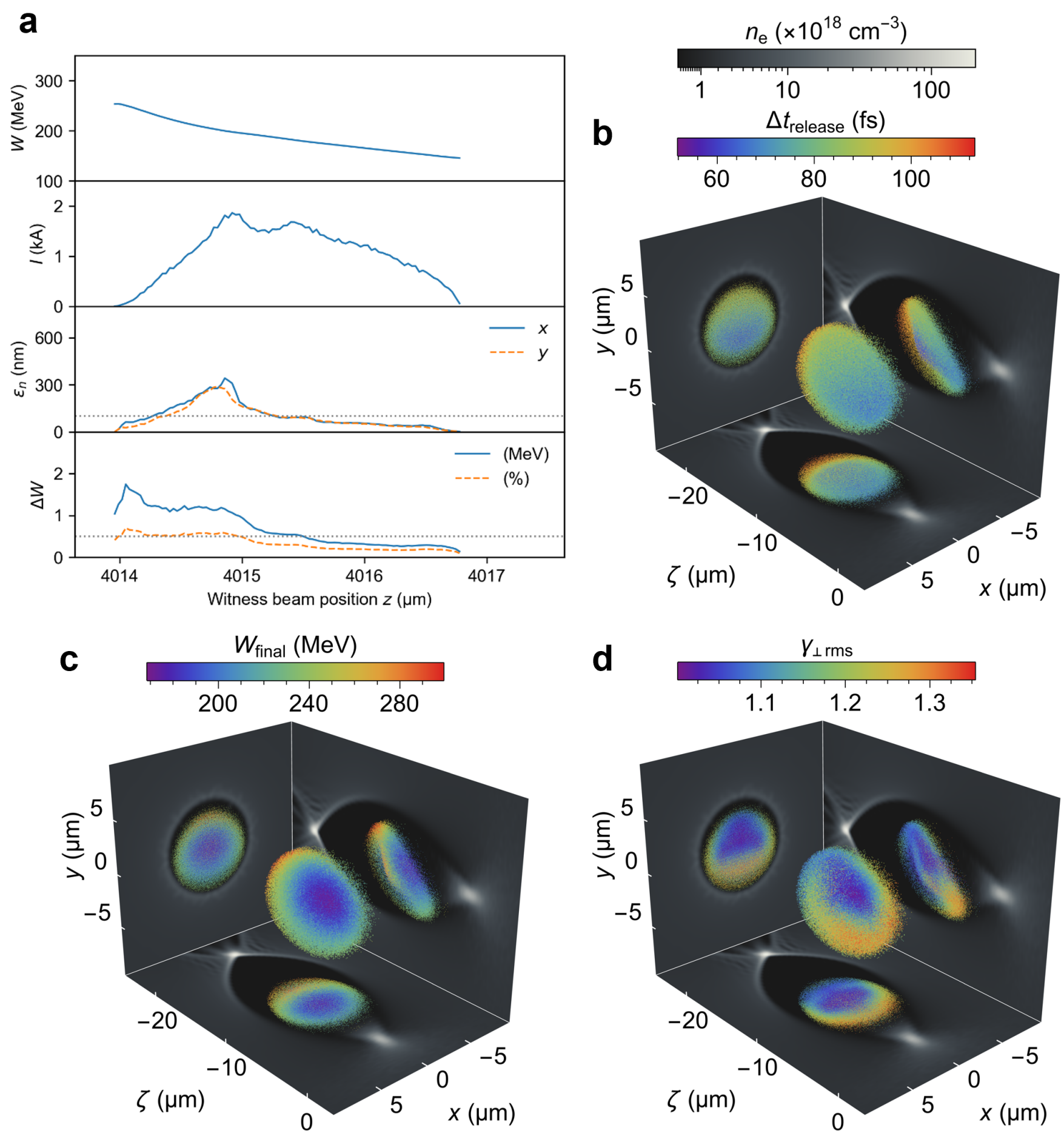

Supplementary Figure 4: Example witness beam slice and source analysis. Representative VSim simulation for a plasma photocathode injector delay of 160 fs. **a**, Slice energy, current, normalized emittance and energy spread. **b-d**, Plasma photocathode source volume colour-coded by release time, final energy and transverse momentum evolution respectively.

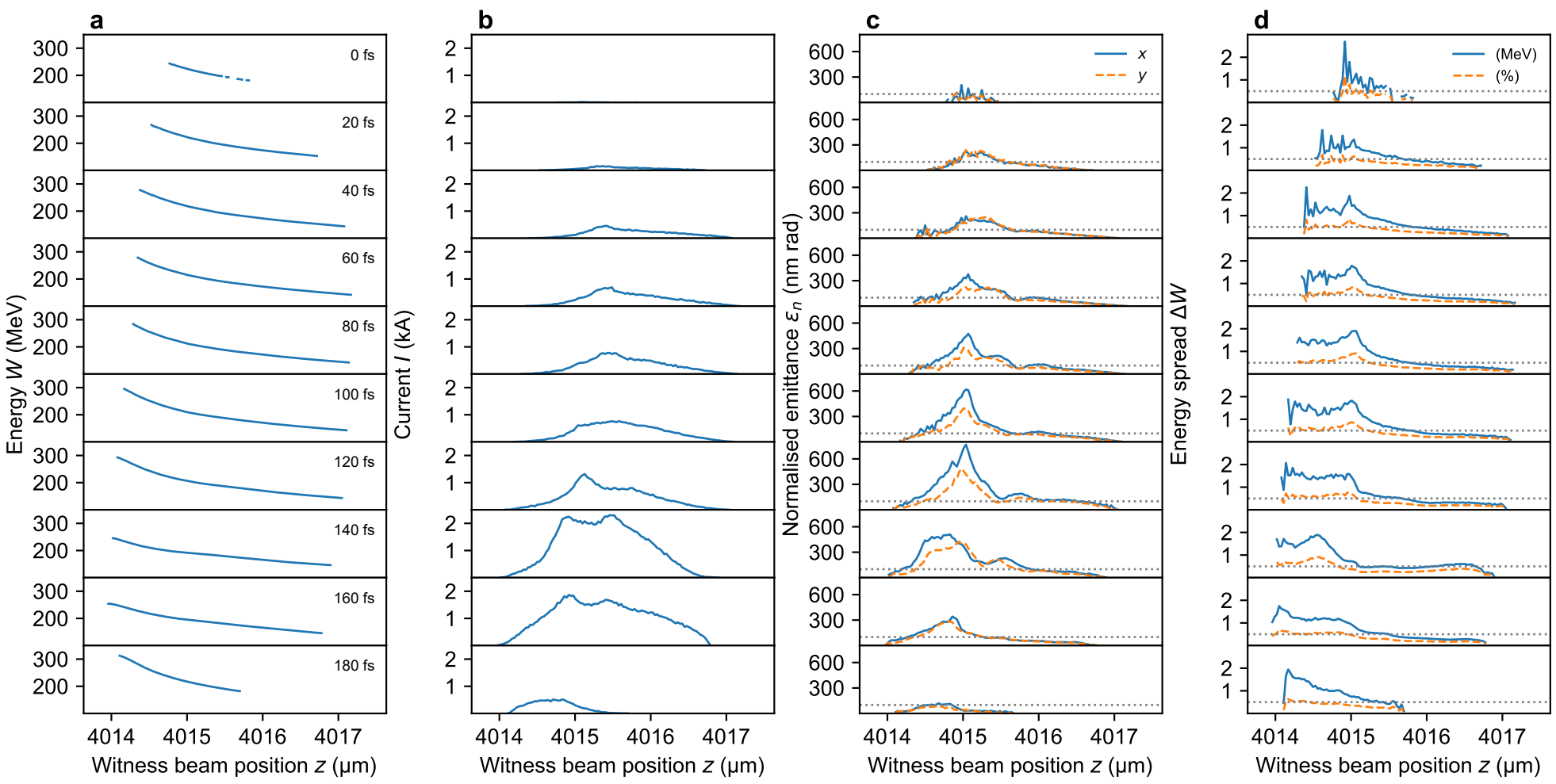

Supplementary Figure 5: Simulation-based witness beam slice analysis. **a**, Mean energy. **b**, Current. **c**, Normalised emittances. **d**, Energy spread. The injector timing for each row is noted in **a**, and the dashed horizontal lines mark the 100 nm·rad emittance and 0.5% energy spread thresholds respectively.

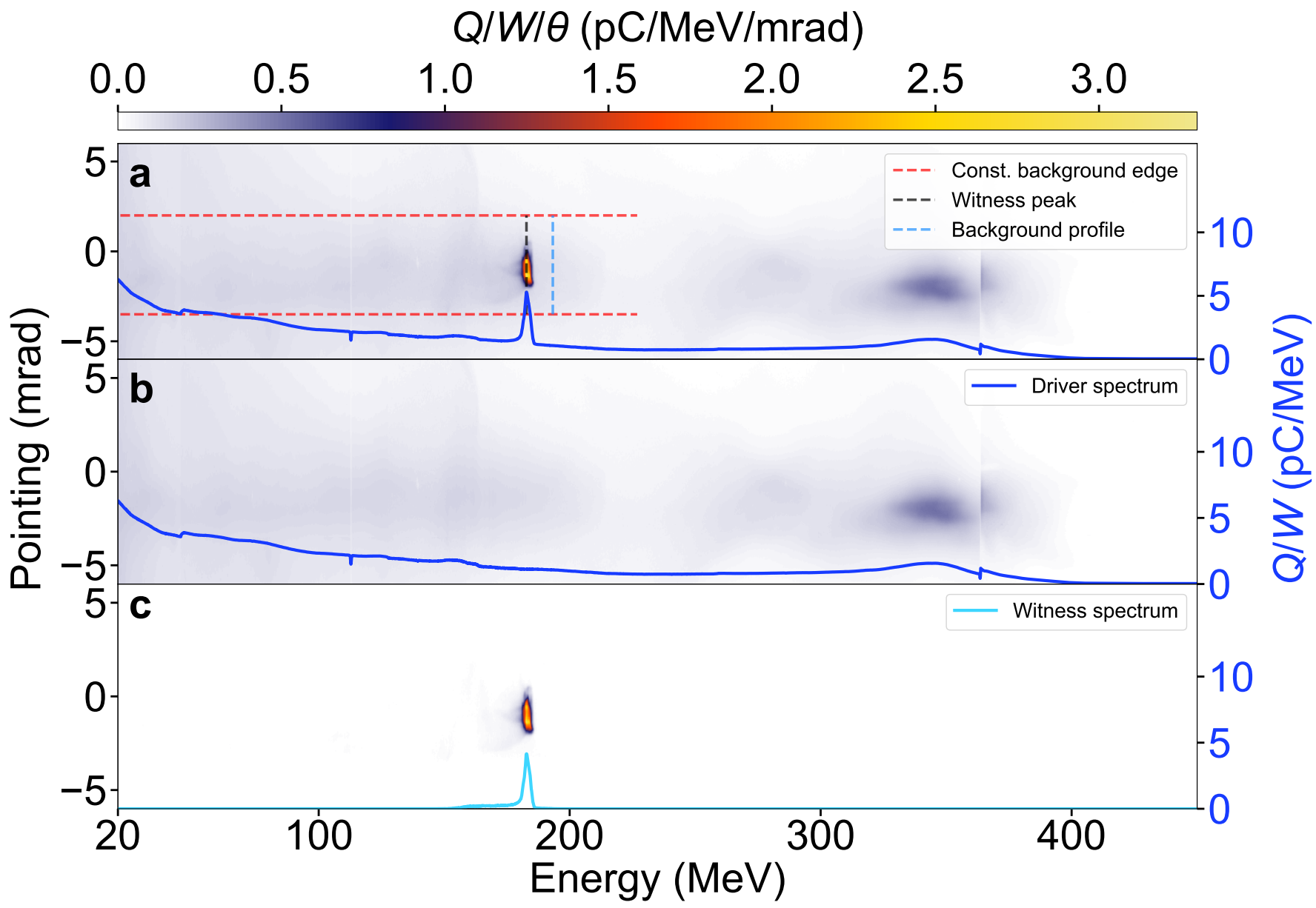

Supplementary Figure 6: Electron spectra Background subtraction **a**, Measured energy spectra with witness and driver. **b**, Separated driver spectrum. **c**, Separated witness spectra.

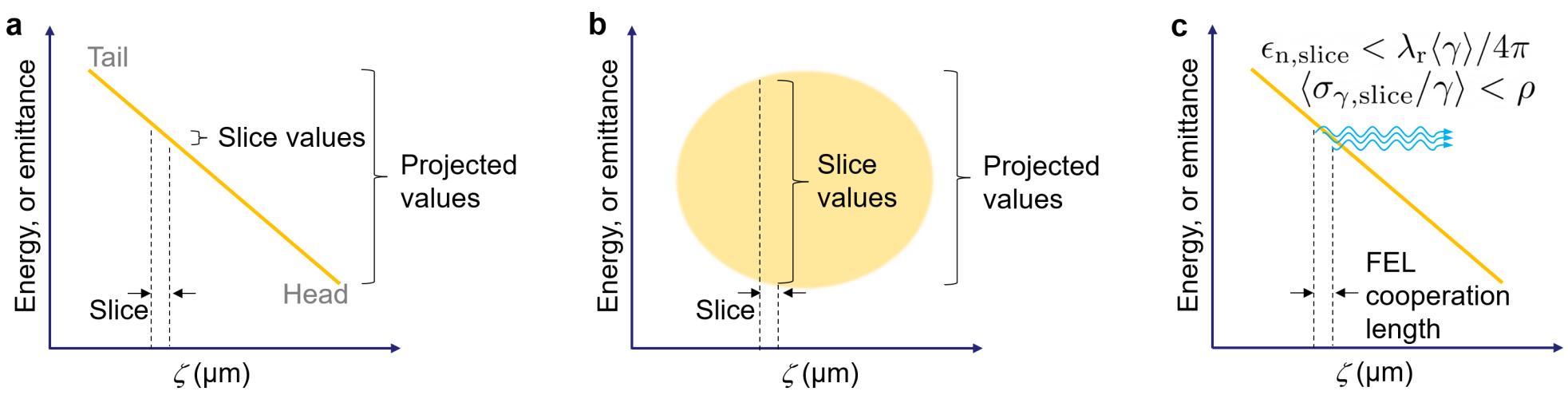

Supplementary Figure 7: Difference of slice and projected beam parameters. **a**, Example of a beam with pronounced chirp, but very small slice beam parameters. **b**, Example of a beam with large slice beam parameters. **c**, Visualization of the importance of slice beam parameters e.g. for free-electron-lasers. Here, the electrons may micro-bunch and emit coherent radiation (blue) from a slice of the cooperation length, but only if slice emittance and slice energy spread are small enough.

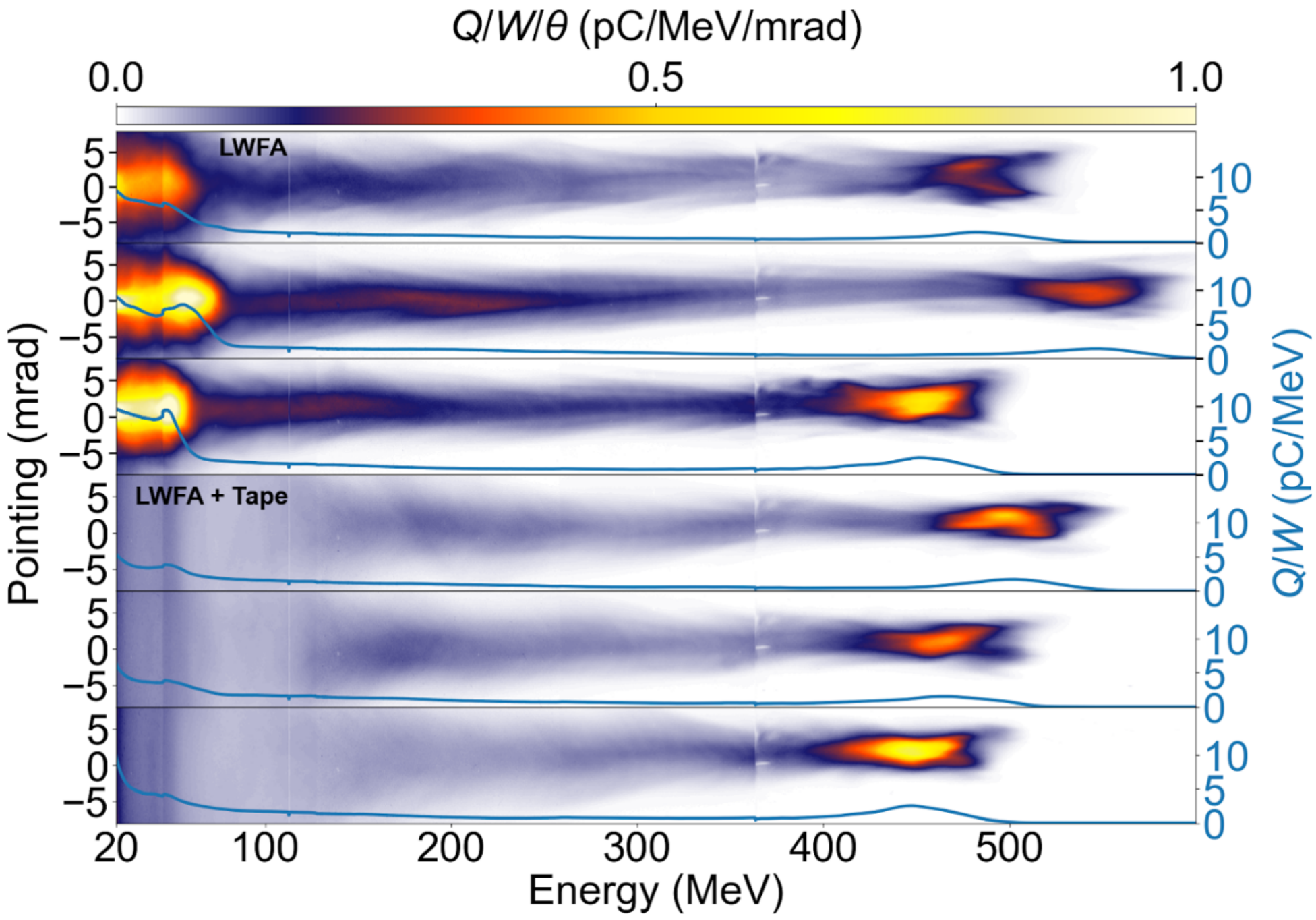

Supplementary Figure 8: LWFA shots with and without tape. The three shots in the top show spectra obtained from LWFA-only shots without the laser blocker tape, the three shots in the bottom show three LWFA-only shots with the laser blocker tape. While the monoenergetic high energy fraction of the electron beam is not notably affected by the tape, scattering cleans up the low energy component of the LWFA-generated beam.

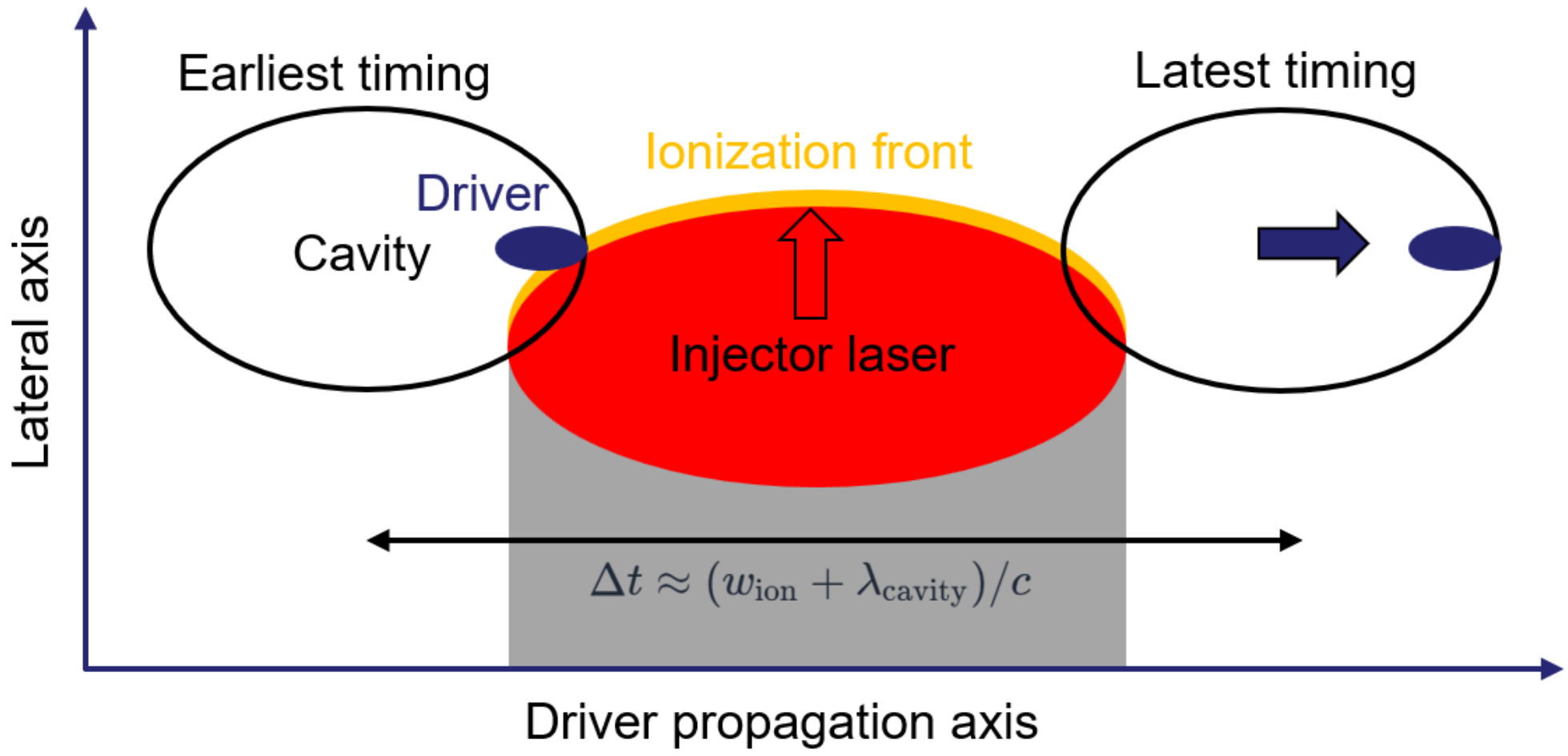

Supplementary Figure 9: Illustration of the injector timing window defined by the relative position of the injector laser (red) and the plasma cavity driven by the electron beam (blue). The two extreme cases correspond to the injector laser overlapping with either the front (left) or the back (right) of the driver-beam cavity. The resulting delay window $\Delta t \approx (w_{\text{ion}} + \lambda_{\text{cavity}})/c$ represents the time required for the driver to propagate the distance between these two configurations. Assuming an ionization-front width of approximately $w_{\text{ion}} \approx 30\,\mu$m and a plasma-cavity length of about $\lambda_{\text{cavity}} \approx 25\,\mu$m, the corresponding temporal window is roughly 180 fs. Experimental jitter, temporal or spatial, as well as the elongation of the first cavity observed will cause an increase in the experimental observed timing window range.

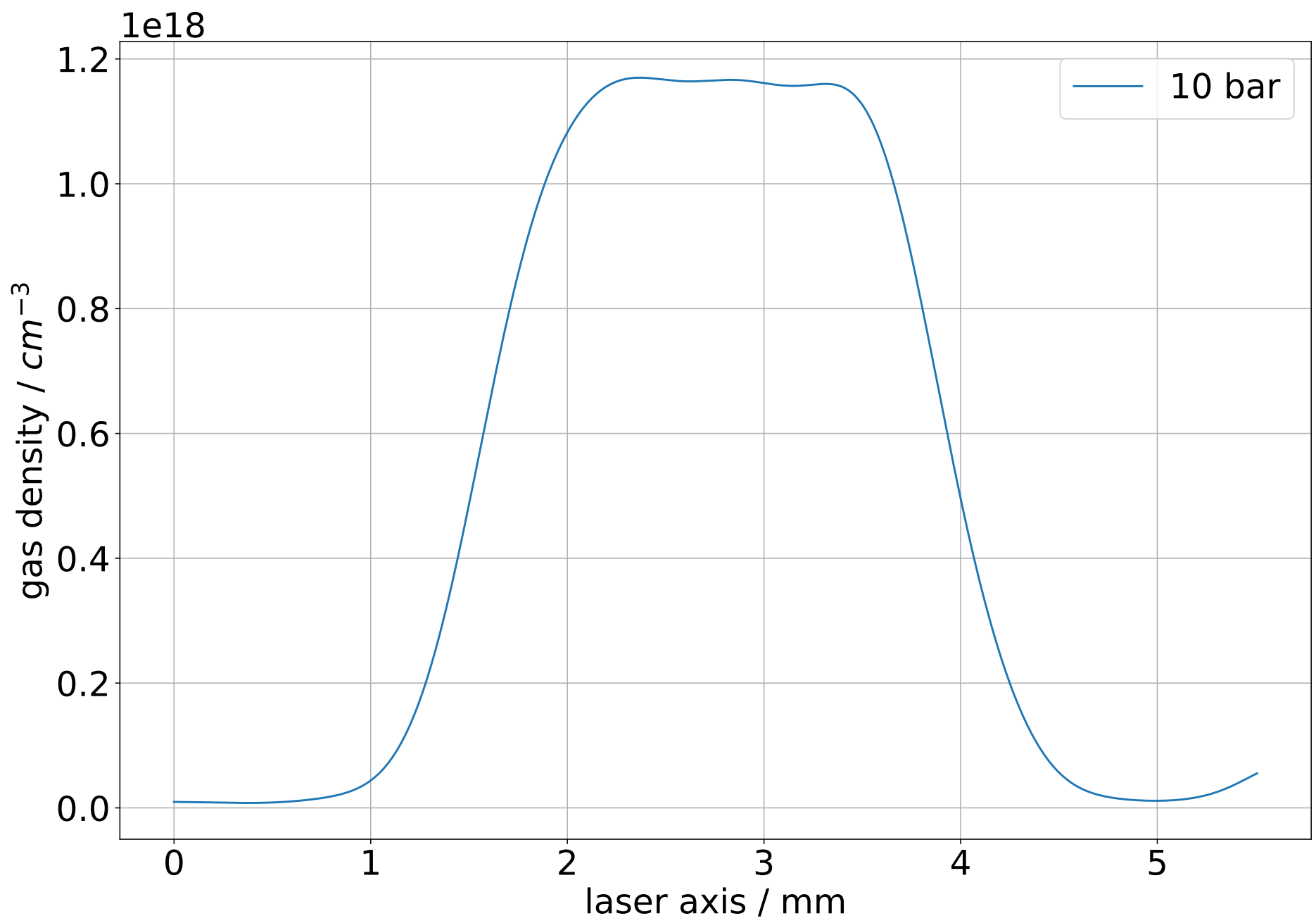

Supplementary Figure 10: Nozzle density profile with the backing pressure used for the experiment (10 bar) measured via interferometry. This profile can be assumed for both stages, as they both consist of a nozzle of this 3-mm-exit target design.

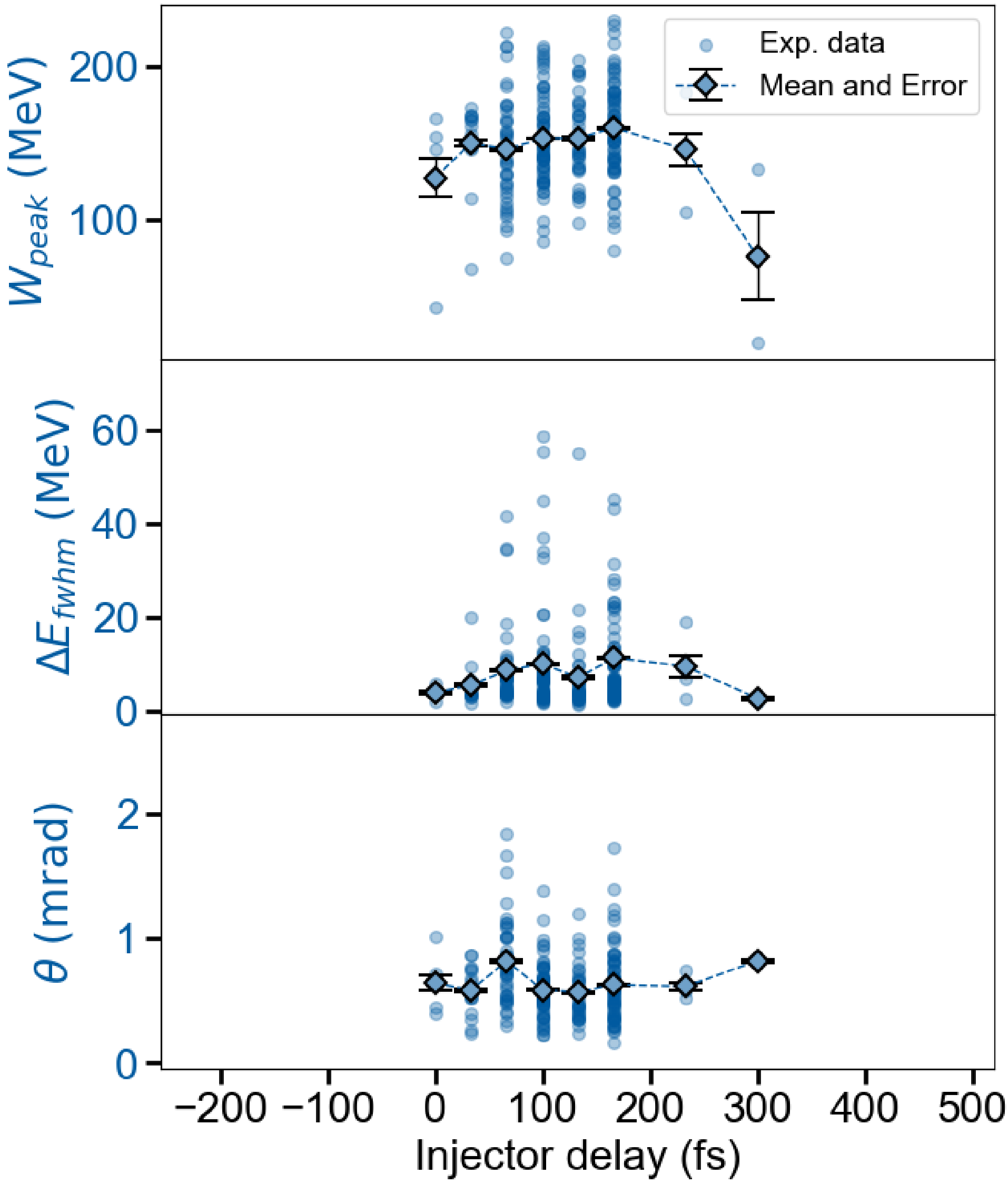

Supplementary Figure 11: Witness beam energy, energy spread, and divergence plots for the timing scan shown in Fig. 3.

References

[1] Rosenzweig, J. B. Nonlinear plasma dynamics in the plasma wake-field accelerator. *Phys. Rev. Lett.* **58**, 555–558 (1987). URL http://link.aps.org/doi/10.1103/PhysRevLett.58.555.

[2] Rosenzweig, J. B., Breizman, B., Katsouleas, T. & Su, J. J. Acceleration and focusing of electrons in two-dimensional nonlinear plasma wake fields. *Phys. Rev. A* **44**, R6189–R6192 (1991). URL http://link.aps.org/doi/10.1103/PhysRevA.44.R6189.

[3] Adli, E. *et al.* Acceleration of electrons in the plasma wakefield of a proton bunch. *Nature* **561**, 363–367 (2018). URL https://doi.org/10.1038/s41586-018-0485-4.

[4] Litos, M. *et al.* High-efficiency acceleration of an electron beam in a plasma wakefield accelerator. *Nature* **515**, 92–95 (2014).

[5] Pompili, R. *et al.* Free-electron lasing with compact beam-driven plasma wakefield accelerator. *Nature* **605**, 659–662 (2022). URL https://doi.org/10.1038/s41586-022-04589-1.

[6] Lindstrøm, C. A. *et al.* Emittance preservation in a plasma-wakefield accelerator. *Nature Communications* **15**, 6097 (2024). URL https://doi.org/10.1038/s41467-024-50320-1.

[7] Habib, A. F. *et al.* Attosecond-angstrom free-electron-laser towards the cold beam limit. *Nature communications* **14**, 1054 (2023).

[8] Rosenzweig, J. B. *et al.* Experimental observation of plasma wake-field acceleration. *Phys. Rev. Lett.* **61**, 98–101 (1988). URL http://link.aps.org/doi/10.1103/PhysRevLett.61.98.

[9] Blumenfeld, I. *et al.* Energy doubling of 42 gev electrons in a metre-scale plasma wakefield accelerator. *Nature* **445**, 741–744 (2007).

[10] Lindstrøm, C. A. *et al.* Energy-spread preservation and high efficiency in a plasma-wakefield accelerator. *Phys. Rev. Lett.* **126**, 014801 (2021). URL https://link.aps.org/doi/10.1103/PhysRevLett.126.014801.

[11] Pompili, R. *et al.* Energy spread minimization in a beam-driven plasma wakefield accelerator. *Nature Physics* **17**, 499–503 (2021).

[12] Rosenzweig, J. B. *et al.* Next generation high brightness electron beams from ultrahigh field cryogenic rf photocathode sources. *Phys. Rev. Accel. Beams* **22**, 023403 (2019). URL https://link.aps.org/doi/10.1103/PhysRevAccelBeams.22.023403.

[13] LaBerge, M. *et al.* Revealing the three-dimensional structure of microbunched plasma-wakefield-accelerated electron beams. *Nature Photonics* (2024). URL https://doi.org/10.1038/s41566-024-01475-2.

[14] Pierce, C. M. *et al.* Low intrinsic emittance in modern photoinjector brightness. *Phys. Rev. Accel. Beams* **23**, 070101 (2020). URL https://link.aps.org/doi/10.1103/PhysRevAccelBeams.23.070101.

[15] Habib, A. F. *et al.* Plasma photocathodes. *Annalen der Physik* **535**, 2200655 (2023).

[16] Manahan, G. *et al.* Single-stage plasma-based correlated energy spread compensation for ultrahigh 6d brightness electron beams. *Nature Communications* **8** (2017). URL https://strathprints.strath.ac.uk/60819/.

[17] Floettmann, K. & Paramonov, V. V. Beam dynamics in transverse deflecting rf structures. *Phys. Rev. ST Accel. Beams* **17**, 024001 (2014). URL https://link.aps.org/doi/10.1103/PhysRevSTAB.17.024001.

[18] Deng, A. *et al.* Generation and acceleration of electron bunches from a plasma photocathode. *Nature Physics* 1–5 (2019).
